# Whole wheat bread improves postprandial glucose tolerance in adults with prediabetes: a controlled-feeding randomized crossover trial with microbiome-associated variation in response

**DOI:** 10.3389/fnut.2026.1907908

**Published:** 2026-08-28

**Authors:** Jillian T. Pierson, Ariana H. Bond, Sisi Cao, Shiqi Zhang, Andrew Gold, Huan Zhang, Kaitlyn M. Zamary, Ayse Gurer, Chao Guo, Menuka Bhandari, Matthew D. Teegarden, Devin G. Peterson, Xiaokui Mo, Jiangjiang Zhu, Richard S. Bruno

**Affiliations:** 1 Human Nutrition Program, The Ohio State University, Columbus, OH, United States; 2 James Comprehensive Cancer Center, The Ohio State University, Columbus, OH, United States; 3 Research and Analytical Core, College of Food, Agricultural, and Environmental Sciences, The Ohio State University, Wooster, OH, United States; 4 Foods for Health Research Initiative, The Ohio State University, Columbus, OH, United States; 5 Department of Food Science and Technology, The Ohio State University, Columbus, OH, United States; 6 Department of Biomedical Informatics, Center for Biostatistics, The Ohio State University, Columbus, OH, United States

**Keywords:** crossover trial, glucose tolerance, gut microbiota, precision nutrition, prediabetes, whole wheat

## Abstract

**Background/hypothesis:**

Whole grain intake is associated with reduced risk of type 2 diabetes, yet inconsistent findings from randomized trials and substantial interindividual variability suggest that gut microbiota may influence glycemic responses. We hypothesized that whole wheat bread would improve glucose tolerance compared with refined white bread under controlled feeding conditions in adults with prediabetes, and that gut microbial features would be associated with variability in glycemic response.

**Methods:**

In this randomized, controlled crossover trial, adults with prediabetes (*n* = 39; 34.7 ± 0.9 kg/m^2^) consumed fully controlled diets differing only in whole wheat (WHEAT) or refined white bread (WHITE) for 2-weeks each, separated by a 2-week washout. Fasting blood and stool samples were collected at baseline and post-intervention, and a 75 g oral glucose tolerance test was performed after each phase.

**Results:**

Compared with WHITE, WHEAT reduced postprandial glucose excursion by 27% (*p* = 0.04), independent of body weight, inflammation, carbonyl stress, and fecal short-chain fatty acids (SCFAs). Fasting glucose and insulin improved over time irrespective of treatment (P_Time_ < 0.05), whereas inflammatory biomarkers, plasma methylglyoxal, and fecal SCFAs did not differ between interventions. Gut microbial *α*- and *β*-diversity were unchanged. However, compositional analyses identified directional shifts in selected taxa during WHEAT, including higher relative abundances of *Agathobacter*, *Agathobaculum*, and *Lachnospiraceae NK4A136 group* and lower *Streptococcus*, *Lachnoclostridium*, and *Mediterraneibacter* (nominal *p* < 0.05). Moderation analysis revealed that baseline abundances of *Lachnoclostridium* and *Lachnospiraceae NK4A136 group* predicted postprandial glycemic responses in a diet-dependent manner, identifying distinct microbial profiles associated with greater metabolic benefit from WHEAT relative to WHITE (P_Interaction_ < 0.05).

**Conclusion:**

Whole wheat bread improves postprandial glucose tolerance in adults with prediabetes while exhibiting microbiome-associated variation in glycemic response. These findings support whole wheat as an effective dietary strategy and highlight baseline gut microbial features as key contributors to inter-individual responses, advancing precision nutrition approaches to whole grain consumption.

**Clinical trials registration:**

ClinicalTrials.gov: NCT05318183.

## 1 Introduction

Prediabetes affects nearly 98 million U.S. adults and substantially increases the risk of type 2 diabetes (T2DM) and other cardiometabolic disorders (1). Given its high prevalence and projection that nearly 25% of U.S. adults will have T2DM by 2050 (2), a critical need exists for dietary strategies that improve glycemic control in individuals at elevated metabolic risk. Among modifiable dietary factors, carbohydrate quality has emerged as an important determinant of glucose homeostasis and cardiometabolic health, including a lower risk of all-cause mortality (3, 4), supporting dietary recommendations that at least half of grain intake derive from whole grains (5).

Whole grain intake has consistently been associated with lower risk of T2DM in prospective cohort studies (6). Compared with refined grains, whole grains retain the bran and germ fractions of the kernel and therefore provide greater amounts of dietary fiber, vitamins, minerals, and phytochemicals that may influence glucose metabolism (7, 8). Whole wheat is among the most commonly consumed whole grains worldwide (9) and is a major source of cereal fiber, especially arabinoxylan, as well as other bioactive compounds, including alkylresorcinols and phenolic acids (8, 10). These constituents may influence glycemia through multiple pathways, including delayed carbohydrate absorption, altered substrate fermentation by the gut microbiota, and interactions with host metabolic signaling.

Despite strong observational evidence linking whole-grain intake to improved glycemic outcomes (3), findings from randomized controlled trials (RCTs) have been inconsistent. For example, a meta-analysis of 37 RCTs (*n* = 3,136 participants) found a modest reduction in fasting blood glucose (−0.103 mmol/L; 95% CI: −0.178 to −0.028), although significant benefits were observed in only 7 of the 37 included trials (6). The lack of agreement between studies could be attributed to several limitations, including inadequate dietary control, significant compositional differences between whole grain types (11), concurrent weight loss that limits understanding of whole grain-specific effects (12, 13), and insufficient study of individuals with prediabetes or other metabolic dysfunction under controlled feeding (14, 15). Consequently, the independent metabolic effects of whole wheat under controlled feeding conditions remain insufficiently defined, particularly in adults at elevated risk for T2DM. A tightly controlled intervention that isolates whole wheat from refined white bread within otherwise matched diets may therefore help establish whether whole wheat improves glucose tolerance in at-risk populations.

The gut microbiota is increasingly recognized as an important factor contributing to interindividual variability in metabolic responses to diet, including whole grains (16, 17). Habitual fiber intake and whole grain consumption are associated with differences in gut microbial composition and function (16), and accumulating evidence suggests that glycemic responses between whole and refined grains can vary substantially between individuals in relation to baseline microbial features (17). Indeed, microbial-derived short-chain fatty acids (SCFAs) have emerged as potential mediators linking dietary fiber utilization to host metabolic regulation and glycemic control (18). Accordingly, the gut microbiota may help explain why some individuals derive greater metabolic benefit from whole grain intake than others. However, whether whole wheat bread improves glucose tolerance in adults with prediabetes under controlled feeding conditions, and whether gut microbial features are associated with variability in metabolic response, has not been well established. In addition to microbiome-related mechanisms, carbonyl stress represents another plausible pathway linking carbohydrate metabolism to metabolic dysfunction (19). Methylglyoxal (MGO), a highly reactive dicarbonyl generated during glycolysis, contributes to protein glycation and has been implicated in insulin resistance and diabetes-related metabolic impairment (19). Although studies suggest that bioactive components of whole grains may attenuate carbonyl stress (19) and that brown rice decreases MGO in adults with prediabetes (20), evidence from controlled human feeding trials examining whole wheat remains limited.

The objective of this study was to determine whether whole wheat bread, incorporated into a rigorously controlled eucaloric diet, improves postprandial glucose tolerance compared with refined white bread in adults with prediabetes. We also examined whether whole wheat consumption alters gut microbiota composition and whether baseline gut microbial features are associated with interindividual variability in glycemic response. In addition, we evaluated inflammatory biomarkers, fecal short-chain fatty acids, and plasma MGO as secondary mechanistic outcomes. We hypothesized that whole wheat bread would improve postprandial glucose tolerance relative to refined white bread and that baseline gut microbiota composition would be associated with variability in glycemic responsiveness. To test this hypothesis, we conducted a randomized, controlled, crossover feeding trial that addresses a critical gap in isolating whole wheat-specific effects under tightly controlled dietary conditions in adults with prediabetes. This investigation is therefore expected to advance an understanding of the metabolic effects of whole wheat and identify microbiome-associated variation in glycemic response.

## 2 Materials and methods

### 2.1 Participants and eligibility

The study design, rationale, and primary objectives of this randomized, controlled, crossover clinical trial have been described previously (21). The protocol was approved by the Biomedical Institutional Review Board at The Ohio State University (2021H0504) and registered at ClinicalTrials.gov (NCT05318183). Participants were recruited from the Columbus, Ohio, USA area between March 2021 and December 2023. All participants provided written informed consent prior to the initiation of study procedures.

Eligibility criteria have been detailed previously (21). In brief, inclusion criteria were adults aged 18–65 years who were overweight or obese (body mass index (BMI) > 27 kg/m^2^) and had prediabetes based on fasting plasma glucose concentrations of 100–125 mg/dL measured under standardized fasting conditions (8–10 h) (22). Exclusion criteria included engagement in aerobic exercise >5 h/week; alcohol consumption ≥2 drinks/day; smoking or vaping; use of dietary supplements containing vitamins, minerals, prebiotics, or probiotics within 1 month of enrollment; or use of medications to manage hyperglycemia, hypertension, or hyperlipidemia. Participants were also excluded if they were pregnant or had a history of gluten intolerance, gastrointestinal or inflammatory disorders, cancer, cardiovascular disease, diabetes, coagulation disorders, or use of antibiotics or antifungals within 6 months prior to enrollment.

### 2.2 Study design

This study consisted of a two-arm, randomized, controlled, crossover design. Eligible participants with confirmed prediabetes were block-randomized using a computer-generated allocation sequence to consume either whole wheat bread (WHEAT) or refined white bread (WHITE) as part of a fully controlled, eucaloric diet for 2 weeks. The treatment order was counterbalanced, and participants served as their own controls. A minimum 2-week washout period, during which participants resumed their habitual diet, separated the intervention arms.

Participants visited the study center every 3–4 days during each intervention period to obtain prescribed foods. Blood pressure and anthropometric measurements were obtained at baseline (PRE; day 1) and post-intervention (POST; day 14). Fasting blood and fecal samples were collected at PRE and POST of each treatment arm for assessment of clinical chemistries, inflammatory biomarkers, carbonyl stress, gut microbiota composition, and microbial metabolites. Fasting blood samples were collected following an overnight fast of 8–10 h, with participants arriving between 0700 and 0900. Whole blood was collected into evacuated tubes with or without ethylenediaminetetraacetic acid as appropriate for biomarker analyses. Serum or plasma was isolated by centrifugation (3,000 × g, 15 min, 4 °C), flash-frozen in liquid nitrogen, and stored at −80 °C until batch analysis.

At POST of each intervention arm, participants completed a 75 g oral glucose tolerance test (OGTT) by ingesting a 500 mL glucose solution that also contained nondigestible sugar probes (lactulose, mannitol, sucralose, erythritol) to concurrently assess intestinal permeability. Blood samples were collected at 30-min intervals for 3 h following glucose ingestion. A complete 24-h urine collection was obtained to assess intestinal permeability; this secondary outcome will be reported separately. The OGTT was administered only at POST to quantify end-of-treatment glucose tolerance while minimizing participant burden associated with repeated glucose challenges. Crossover counterbalancing and washout periods were used to support within-participant inference.

### 2.3 Study duration, bread dose, and formation

The primary objective of this trial was to evaluate WHEAT consumption on improvements in glucose tolerance in adults with prediabetes. Prior RCTs suggest that whole grain–mediated improvements in glycemia may occur within as little as 2 weeks (23). Accordingly, a 2-week intervention period with rigorous dietary control was selected to assess early metabolic responses and potential prebiotic activities associated with wheat consumption.

During each intervention period, participants consumed test breads (160 g/day) as part of either WHEAT or WHITE, consistent with the Dietary Guidelines for Americans (DGA), which recommend that whole grains comprise at least half of total grain intake (5). The whole wheat bread met the USDA definition of a whole grain, retaining the bran, germ, and endosperm in their original proportions following milling (24). The test breads were produced under standardized conditions at the Ohio State University Food Industries Center to ensure uniformity and frozen at −20 °C until used. Nonfortified whole wheat and white flours were sourced from the same distributor (Ardent Mills). Breads were prepared using the straight-dough method (25), and proximate composition was performed by Eurofins Nutrient Analysis Center (21). The use of minimally formulated test breads, combined with a fully controlled background diet, minimized confounding and enabled isolation of the glucose-lowering effects attributable specifically to the endogenous components of whole wheat flour.

### 2.4 Dietary control

To minimize dietary heterogeneity and isolate treatment effects attributable to the test breads, participants consumed a fully controlled diet during each intervention period. Diets were identical between treatment arms except for the type of bread provided. To support interpretability, diets were matched for energy and macronutrient distribution. The principal planned difference between arms was the substitution of whole wheat versus refined flour bread, which necessarily altered total dietary fiber and endogenous grain constituents. Participant energy requirements were estimated using the Harris–Benedict equation and adjusted for physical activity, a well-established and widely used method for estimating energy requirements in free-living adults that incorporates sex-specific prediction equations (26). Body mass was monitored weekly, and prescribed energy intake was adjusted when body mass deviated by more than 1.5 kg from PRE to help maintain energy balance throughout the intervention. Diets were prescribed using a 4-day rotating menu at energy intakes ranging from 1,800 to 3,300 kcal/day in 200–300 kcal increments based on individual needs (Supplementary Table 1). Portion sizes were adjusted accordingly.

Controlled diets were designed to provide 50–52% of energy from carbohydrate, 30–33% from fat, and 18–19% from protein, consistent with typical U.S. macronutrient distributions (27). Diets were devoid of whole grains, fermented foods, and probiotic-containing foods. Diets were intentionally not matched for fiber to preserve the native fiber composition of whole wheat. WHITE provided 8–10 g fiber/1,000 kcal, consistent with the American diet (28), whereas WHEAT provided 10–12 g fiber/1,000 kcal. Foods provided on the final day of each intervention during the 24-h urine collection were free of sugar alcohols (Supplementary Table 1). Participants were instructed to consume only the food provided and to document any deviations. All uneaten food or empty packaging was returned and weighed to assess compliance, and bread counts were used as an additional compliance metric. Energy and nutrient intakes were analyzed using the Nutrition Data System for Research (University of Minnesota). Diet quality was assessed using the Healthy Eating Index-2020 (HEI-2020) to quantify alignment with the DGA (29).

### 2.5 Clinical chemistries

Plasma glucose (G75171L), triglyceride (T7532500), total cholesterol (C7510500), and HDL-cholesterol (H751160) were measured using spectrophotometric clinical assays (Pointe Scientific) on a Shimadzu UV-2600 spectrophotometer. LDL-cholesterol was calculated using the Friedewald equation (30). Insulin was measured by ELISA on a Synergy H1 microplate reader (Biotek Instruments) according to the manufacturer’s instructions (80-INSHU-E01.1, E10.1; Alpco). All assays were performed in duplicate. Intra- and inter-assay coefficients of variation of assay controls were 4–12%.

Insulin resistance and insulin sensitivity were estimated from fasting glucose and insulin concentrations using the homeostatic model assessment of insulin resistance (HOMA-IR) and the quantitative insulin sensitivity check index (QUICKI) (31). OGTT-derived indices of insulin sensitivity and β-cell function were calculated from glucose and insulin concentrations, which included the Matsuda-DeFronzo Insulin Sensitivity Index (31), Insulinogenic Index (32), Oral Disposition Index (33), and Stumvoll Index (34).

### 2.6 Intestinal and host inflammation

Fecal calprotectin (Alpco, 30-CALPHU-E01) and myeloperoxidase (MPO; Immundiagnostik, KR6630) concentrations were measured using ELISA kits to assess intestinal neutrophil infiltration and activity, respectively, as described (35). Systemic inflammation was assessed by measuring serum C-reactive protein (CRP; DCRP00B), interleukin-6 (IL-6; HS600C), and tumor necrosis factor-*α* (TNF-α; DTA00D) using ELISA kits (R&D Systems) according to the manufacturer’s instructions. Samples from both periods for a given participant were analyzed on the same plate/batch to minimize batch effects.

### 2.7 Fasting and postprandial MGO

Fasting and postprandial MGO were measured to assess basal and OGTT-induced carbonyl stress. Plasma MGO was quantified by LC–MS, as described in (36), with minor modifications. The internal standard, tetradeuterated-2-methylquinoxaline (d_4_-2-MQ), was synthesized from unlabeled methylglyoxal (d_0_-MGO) via derivatization with octadeuterated-o-phenylenediamine (d_8_-oPD; Cambridge Isotopes) (36). In brief, plasma (30 μL) was incubated with 90 μL of d_0_-oPD (9.25 mM in 1.6 M perchloric acid) for 24 h at 22 °C in the dark to generate d_0_-2-MQ. Following derivatization, d_4_-2-MQ (5 μL; 5.2 μM) was added, and samples were centrifuged (16,000 × g, 30 min, 4 °C) prior to filtering the supernatant through a 0.22 μm PVDF filter (Merck Millipore). Filtrate (5 μL) was analyzed on a Shimadzu LCMS-2020 operated in positive electrospray ionization mode. Chromatographic separation was achieved at 30 °C on an Acquity UPLC BEH C18 column (2.1 × 50 mm, 1.7 μm; Waters Corp) at a flow rate of 0.4 mL/min using a binary gradient of 0.01% formic acid in water (A) and acetonitrile (B). Initial gradient conditions were 1% mobile phase B, followed by a linear gradient to 25% over 8 min, an increase to 50% mobile phase B over 1 min, and an isocratic hold for 2 min. Initial conditions were then restored over 1 min and held for 2 min before the next injection. Mass spectra were acquired using an interface voltage of 4.5 kV, a detector voltage of 1.55 kV, a heat block temperature of 400 °C, and a desolvation gas temperature of 250 °C. Nitrogen was used as the nebulizer gas (1.5 L/min) and drying gas (15 L/min). Analytes were quantified by single-ion monitoring using peak-area ratios relative to the internal standard at m/z 145.1 (d_0_-2-MQ) and 149.1 (d_4_-2-MQ).

### 2.8 Fecal short-chain fatty acids

Fecal SCFAs, including straight- and branched-chain species, were quantified by LC–MS following 3-nitrophenylhydrazine derivatization, as described (37), with minor modifications (35). In brief, fecal samples were homogenized, centrifuged, and the resulting supernatant was spiked with the internal standard ^13^C₄-butyrate (Cambridge Isotope Laboratories) prior to derivatization with 3-nitrophenylhydrazine to generate corresponding 3-nitrophenylhydrazine-SCFA derivatives. Samples were analyzed by LC–MS, with binary gradient chromatographic separation performed at 0.35 mL/min using an Acquity UPLC BEH C18 column (2.1 × 100 mm, 1.7 μm; Waters Corp). Analytes were detected at their respective mass-to-charge ratios and quantified using peak area ratios relative to the internal standard.

### 2.9 Fecal DNA extraction and sequencing

Fecal samples collected at PRE and POST were used to characterize gut microbial community structure. Total DNA was extracted using a repeated bead-beating plus column method (QIAamp DNA Stool Mini Kit; Qiagen) (21), with minor modifications. Briefly, samples underwent combined chemical and mechanical lysis using a Bead Mill 24 Homogenizer (Fisher Scientific) at maximum speed for 3 min. Following precipitation and removal of RNA and proteins, DNA was washed with ice-cold ethanol to improve purity. DNA concentration and purity were assessed using a NanoDrop OneC spectrophotometer (Thermo Fisher Scientific). Extracted DNA was submitted to the Molecular and Cellular Imaging Center at The Ohio State University for 16S rRNA gene amplicon sequencing targeting the V3–V4 hypervariable region using primers 341F (CCTACGGGNGGCWGCAG) and 785R (GACTACHVGGGTATCTAATCC). Normalized DNA samples underwent PCR amplification and indexing using the Nextera XT Index Kit (Illumina). Pooled libraries were sequenced on an Illumina MiSeq platform using a 2 × 301 bp paired-end run.

### 2.10 Microbiota bioinformatics and analysis

Computation was performed at the Ohio Supercomputer Center at Ohio State University. Raw demultiplexed FASTQ files were quality-checked using FastQC (v0.12.1) and MultiQC (v1.29), followed by primer removal with cutadapt (v5.1). Four samples were excluded due to low sequencing depth (<500 reads), yielding n = 152 for downstream processing. Downstream analyses were conducted in R (v4.2.3) and Miniconda3-managed conda environments. Amplicon sequence variants (ASVs) were inferred using DADA2 (v1.16) (38) with standard processing steps: quality filtering and trimming, error model learning, denoising, merging paired reads, length filtering (retaining ASVs 400–445 bp), and chimera removal. Forward and reverse reads were truncated at 265 bp and 192 bp, respectively. Truncation length was selected after inspecting the per-base quality profile generated using plotQualityProfile() function in DADA2. Following the standard DADA2 recommendation, reads were truncated when the Phred quality decline while maintaining sufficient overlap for paired-end merging (38). Given an expected amplicon length of 444 bp, the selected forward and reverse truncation lengths exceed the minimum 12-bp overlap required by DADA2 for successful merging. Taxonomy was assigned using the SILVA database (v138.2) (39). Sequencing generated a mean of 31,430 reads per sample (min 5,340; max 62,597), with 10,463 reads per sample remaining after filtering (min 3,909; max 30,391).

To construct a phylogenetic tree, sequences were aligned with MAFFT (v7.526) (40), and a tree was constructed using FastTree (v2.2.0) (41). Count tables and taxonomy generated from DADA2, phylogeny, and metadata were integrated into a single phyloseq object. Rarefaction curves were generated using the vegan package in R (v2.6–4), and samples were rarefied to 5,000 reads for diversity analyses. Two samples were excluded after rarefaction due to insufficient depth, yielding n = 150 for diversity analyses. Rarefaction was applied only for *α*- and β-diversity calculations, whereas differential abundance and functional analyses used non-rarefied data with centered log-ratio (CLR) transformations. α-Diversity metrics (Observed Features, Chao1, Shannon) were calculated and compared between treatments using paired tests appropriate for the crossover design (Wilcoxon signed-rank tests). β-Diversity was assessed using weighted and unweighted UniFrac distances. Group separation was visualized by PCoA, and differences were tested using PERMANOVA with permutations constrained within participant, consistent with the crossover design. Functional potential was inferred from ASVs using PICRUSt2 via the picrust2_pipeline.py workflow (42). Prediction confidence was evaluated using NSTI, and NSTI distributions were reviewed as part of quality control. Because functional profiles are inferred from ASVs, PICRUSt2 outputs were treated as exploratory and interpreted conservatively as predicted functional capacity. Predicted genes were annotated using KEGG Orthologs to infer functional pathways, which were collapsed into KEGG pathways using ggpicrust2 in R (43).

### 2.11 Statistical analysis

The predefined primary outcome of this RCT focuses on responder stratification and will be reported separately (21); therefore, all analyses presented here are secondary. For this report, our objective was to determine whether adults with prediabetes exhibit improved glucose tolerance in response to WHEAT compared with WHITE based on postprandial glucose responses during the OGTT. Data were analyzed using R (v4.2.3) and GraphPad Prism 11. Prior to statistical testing, data distributions were evaluated to assess normality assumptions. When necessary, data were log-transformed to satisfy model assumptions. Following assessment and transformation, all biochemical outcomes met normality assumptions and were analyzed using parametric statistical methods. Statistical significance for all analyses was defined as *p* ≤ 0.05.

For clinical, metabolic, and inflammatory outcomes measured at PRE and POST, data were analyzed using 2-way ANOVA with repeated measures using time (PRE/POST) and treatment (WHEAT/WHITE) as within-participant factors. When significant time x treatment interactive effects were detected, post-hoc comparisons with Bonferroni’s adjustment were performed. Postprandial variables assessed at POST only, including glucose, insulin, and MGO, were calculated as the change in concentration from baseline (t = 0 min) followed by the trapezoidal rule to determine AUC_0-180 min_. Within-participant comparisons between WHEAT and WHITE were evaluated using paired t-tests. Associations between variables, accounting for within-participant repeated measures, were evaluated using repeated-measures correlation (rmcorr).

Differential abundance testing of CLR-transformed data (pseudo-count 0.01) was performed at the phylum and genus levels after applying filters for total abundance (>0.5%) and prevalence (>10%). Analysis was performed using linear mixed models (LMM; R packages lmer and lmertest) with WHEAT as the reference treatment. The initial models included fixed effects for treatment, time, time × treatment, age, sex, BMI, and treatment order, with participants as a random effect. Only time, treatment, and their interaction were retained as fixed effects in the final models, as other covariates did not explain additional variation or improve model fit. Multiple testing was controlled using the Benjamini–Hochberg false discovery rate (FDR). Taxa-level results not subjected to FDR correction were interpreted as exploratory and hypothesis-generating. Nominal *p*-values and FDR-adjusted Q-values are reported.

Moderation analyses were conducted to examine whether baseline (PRE) gut microbiota abundance modified postprandial glycemic responses to dietary treatment. For these analyses, postprandial glucose and insulin AUCs were modeled using regression frameworks that included baseline (PRE) CLR-transformed taxa abundance, treatment, and their interaction. A statistically significant taxa × treatment interaction was interpreted as evidence of moderation. Additional exploratory analyses examined associations between post-intervention (POST) CLR-transformed taxa abundance and postprandial glycemic outcomes within each dietary condition. Moderation analyses were restricted to taxa demonstrating significant time, treatment, or interaction effects in initial differential abundance testing.

## 3 Results

### 3.1 Participant flow and baseline characteristics

**Figure 1:**
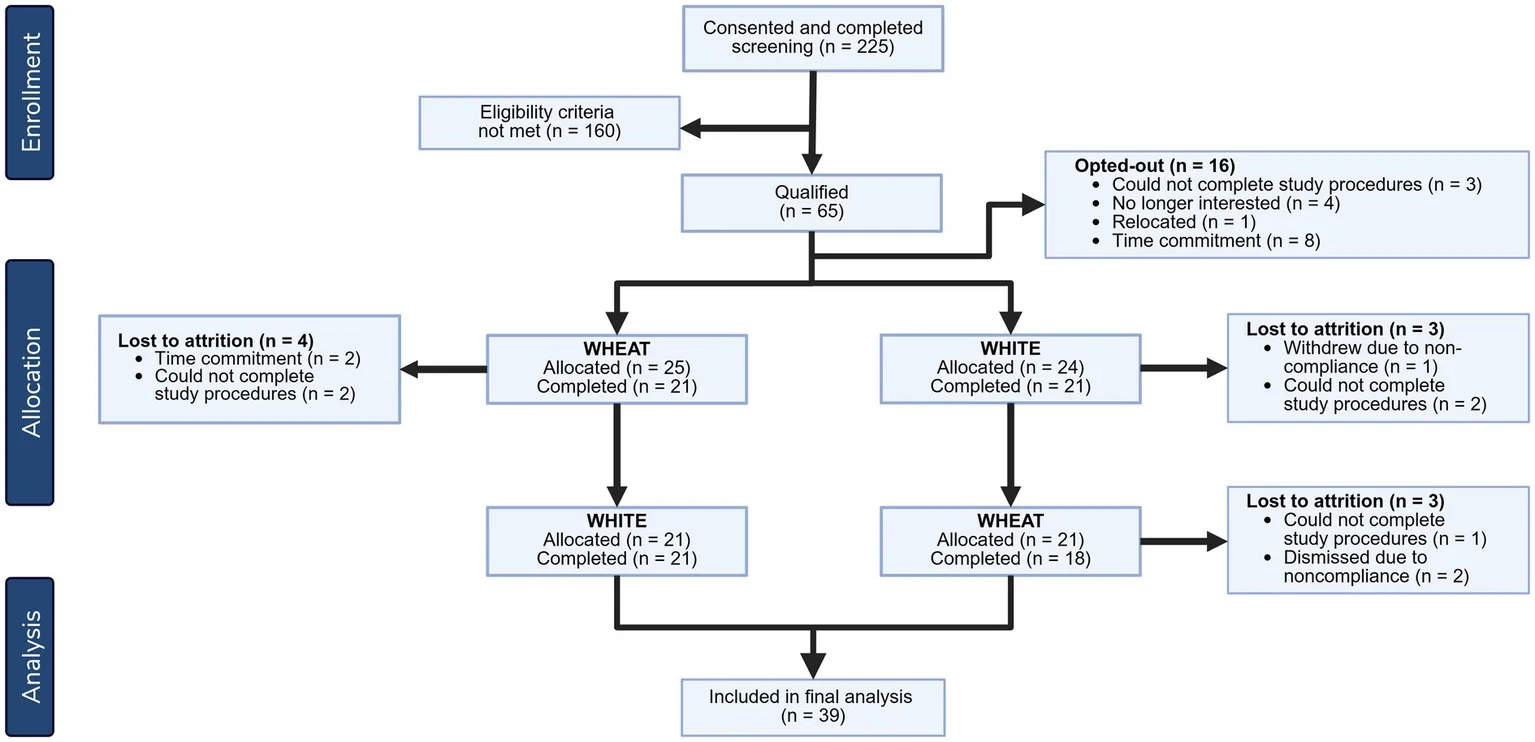
CONSORT diagram illustrating participant flow in a randomized crossover trial evaluating whole wheat and refined white bread consumption on cardiometabolic health in adults with prediabetes. After screening, eligible participants with confirmed prediabetes were randomized to a 2-week intervention with either whole wheat (WHEAT; 160 g/day) or white bread (WHITE) consumed within a controlled diet. Of 65 eligible participants, 49 were randomized, and 39 completed both intervention phases and were included in the final analysis.

A total of 65 adults with prediabetes fulfilled the study criteria and were eligible for enrollment (Figure 1). Of these, 16 participants withdrew before randomization for personal reasons (e.g., time commitment, disinterest). Seven participants were lost to follow-up during the first study period. In the second study period, one participant was lost due to an inability to complete study procedures, and two were dismissed from the study for noncompliance with the prescribed diet. In total, 39 participants (21 men and 18 women; Table 1) completed all study procedures and were included in the final data analysis.

**Table 1:** Baseline characteristics of participants with prediabetesa, b, c.

Characteristic	All (*n* = 39)	Male (*n* = 21)	Female (*n* = 18)
Age (y)	40.5 ± 2.0	39.5 ± 1.9	41.7 ± 2.6
BMI (kg/m^2^)	34.7 ± 0.9	33.5 ± 0.9	36.0 ± 1.3
Waist Circumference (cm)	106.1 ± 2.2	108.5 ± 2.0	103.1 ± 3.0
SBP (mmHg)	122.6 ± 1.8	123.5 ± 1.7	121.7 ± 1.7
DBP (mmHg)	81.9 ± 1.3	80.2 ± 1.3	83.9 ± 1.5
Fasting Plasma Glucose (mg/dL)	108.7 ± 1.1	107.4 ± 1.0	110.1 ± 1.5

Parameters were assessed at screening prior to the completion of a randomized, controlled, crossover trial examining 2-week consumption of a fully prescribed diet containing either refined bread or wheat bread (160 g/d).

^a^ Data are means ± SEM.

^b^ Between-sex comparisons were performed using unpaired Student’s *t*-test. No statistically significant differences by sex status were observed (*p* > 0.05).

^c^ BMI, body mass index, DBP, diastolic blood pressure, SBP, systolic blood pressure.

Participants’ baseline characteristics (Table 1) confirmed the presence of prediabetes based on fasting glucose concentrations. On average, participants were classified as obese based on BMI (34.7 ± 5.1 kg/m^2^), with 23% categorized as overweight (25–30 kg/m^2^) and 77% as obese (30–40 kg/m^2^). Most male participants (90%) and all female participants (100%) met sex-specific waist circumference criteria for central obesity (21). Although 23% of the cohort was normotensive, 77% had elevated blood pressure or hypertension; no participants were using antihypertensive medications, consistent with study exclusion criteria. No significant differences were observed between sexes for any baseline characteristics (*p* > 0.05).

### 3.2 Dietary compliance: test breads and prescribed diets

Study compliance was evaluated based on consumption of the test breads (160 g/day) and adherence to the prescribed diets. Analysis of weighed food records indicated that participants consumed 98.8 and 99.7% of total test bread servings during the WHITE and WHEAT phases, respectively, with no difference between treatments (*p* > 0.05; Table 2). Participants also demonstrated high compliance with prescribed dietary intakes, consuming 97–98% of provided energy during both study arms.

**Table 2:** Dietary analysis from participants with prediabetes who completed the randomized, controlled crossover trial examining daily consumption of 160 g/d of wheat bread or refined bread as part of a fully prescribed diet.^1^

Dietary parameter	White	Wheat	*P* ^2^
Bread consumed (% of total provided)^3^	98.8 ± 0.01	99.7 ± 0.0	0.11
Prescribed energy (kcal)^4^	2,651 ± 73	2,612 ± 65	-
Consumed energy (kcal)^4^	2,533 ± 69	2,567 ± 61^5^	0.43
Carbohydrate (% kcal)	51.0 ± 0.2	49.8 ± 0.2	<0.0001
Dietary fiber (g)	24.6 ± 0.6	31.4 ± 0.6	<0.0001
Insoluble fiber (g)	15.2 ± 0.5	22.6 ± 0.4	<0.0001
Soluble fiber (g)	7.2 ± 0.6	7.6 ± 0.20	0.001
Fat (% kcal)	31.0 ± 0.2	31.9 ± 0.2	0.88
Protein (% kcal)	18.0 ± 0.2	17.9 ± 0.1	0.19
Non-prescribed energy (% kcal)	2.3 ± 0.5	2.4 ± 0.7	0.80
Total HEI-2020 score (0–100)^5^	54.3 ± 0.4	69.7 ± 0.5	<0.0001
HEI-2020 adequacy components			
Total fruits (0–5)	3.4 ± 0.1	3.4 ± 0.1	0.85
Whole fruits (0–5)	4.0 ± 0.2	4.0 ± 0.2	0.54
Total vegetables (0–5)	3.4 ± 0.1	3.4 ± 0.1	0.63
Greens and beans (0–5)	1.8 ± 0.1	1.8 ± 0.1	0.22
Whole grains (0–10)	1.7 ± 0.1	9.9 ± 0.0	<0.0001
Dairy (0–5)	3.8 ± 0.1	3.8 ± 0.1	0.92
Total protein foods (0–5)	4.9 ± 0.0	4.9 ± 0.0	0.62
Seafood and plant proteins (0–5)	3.2 ± 0.1	3.3 ± 0.1	0.25
Healthy fats (0–10)	5.4 ± 0.1	5.5 ± 0.2	0.40
HEI-2020 moderation components			
Refined grains (0–10)	1.6 ± 0.1	8.3 ± 0.1	<0.0001
Sodium (0–10)	4.5 ± 0.2	4.6 ± 0.1	0.65
Added sugars (0–10)	9.0 ± 0.1	9.0 ± 0.1	0.69
Saturated fat (0–10)	7.5 ± 0.1	7.6 ± 0.1	0.05

1 Dietary analysis includes all of the participants’ dietary intake during both 14-day intervention periods, including any foods consumed outside the diet.

2 Data (means ± SEM, *n* = 39) were analyzed using a paired Student’s *t*-test.

3 Bread consumption calculated from the number of bread servings consumed relative to the servings allocated.

4 Participants’ prescribed energy was based on the Harris-Benedict equation, adjusted for physical activity. Consumed energy was calculated from daily weighed food records during each 14-day intervention period.

5 Healthy Eating Index (HEI-2020) calculated based on participants’ complete dietary intakes, including test bread. HEI-2020 Component scores are weighted from 0–5 or 0–10 points, with adequacy components improving the total score and moderation components reducing it.

**Figure 2:**
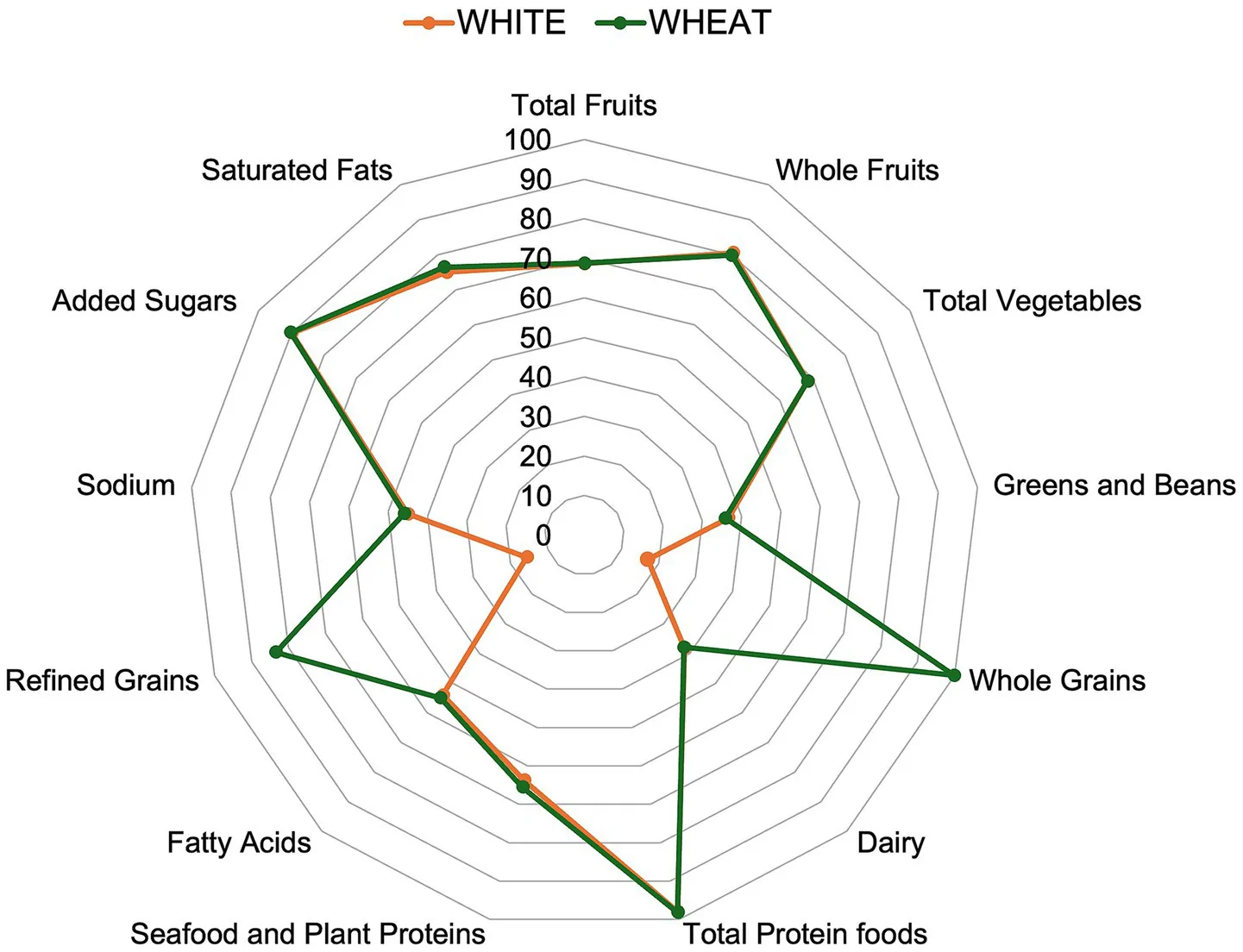
Radar plot of Healthy Eating Index–2020 (HEI-2020) component scores illustrating diet quality in participants who completed a randomized, controlled crossover trial examining the effects of whole wheat and refined white bread consumption on cardiometabolic health. Participants (*n* = 39) consumed 160 g/day of either refined white bread (WHITE) or whole wheat bread (WHEAT) as part of a fully prescribed diet for 2 weeks. HEI-2020 component scores were calculated as (intervention component score/maximum possible score) × 100. Dietary analysis indicated that the controlled diets were well matched in overall quality, differing primarily by the type of test bread provided.

Total energy, fat, and protein intakes did not differ between treatments (*p* > 0.05; Table 2). However, carbohydrate intake was modestly lower during WHEAT compared with WHITE (49.8% vs. 51.0% kcal; *p* < 0.0001). Total dietary fiber intake was significantly higher during WHEAT compared with WHITE (31.4 vs. 24.6 g/day; *p* < 0.0001), consistent with the planned study design. This increase was primarily attributable to non-fermentable fiber (*p* < 0.0001), consistent with proximate composition analyses indicating ~6.7 g/day fiber contribution from whole wheat bread (21). HEI-2020 scores during WHITE (54.3 ± 0.4) were consistent with the typical U.S. diet (58 points) (44), whereas HEI-2020 scores were 23% higher during WHEAT (69.7 ± 0.5; *p* < 0.0001; Table 2). Analysis of HEI subcomponents (Table 2) and radar plot visualization (Figure 2) indicated that this difference was driven by increased whole grain intake and reduced refined grain intake. Overall, adherence to the test breads and prescribed diets was high across study arms.

### 3.3 Cardiometabolic health

No significant time × treatment interactions were observed for BMI, blood pressure, or blood lipids between the intervention arms (*p* > 0.05; Table 3). However, a significant main effect of time indicated reductions in BMI at POST (−0.27 kg/m^2^; *p* < 0.0001), which corresponded to a mean decrease in body mass of 0.83 kg. Diastolic (−2.6 mmHg, *p* = 0.0013) and systolic (−2.7 mmHg, *p* = 0.0087) blood pressure also decreased over time independent of treatment. Systolic blood pressure was modestly lower during WHEAT compared with WHITE (*p* = 0.03), independent of time. Blood lipids (total, LDL-C, triglyceride, and HDL-C) were unaffected by time and treatment (*p* > 0.05). These findings indicate modest improvements in body weight and blood pressure that were independent of bread type.

**Table 3:** Clinical and fasting cardiometabolic biomarkers of persons with prediabetes at the beginning and end of each treatment arm who completed the randomized, controlled crossover trial examining 2-week consumption of either 160 g/d of whole wheat bread or white bread as part of a fully prescribed diet.

Cardiometabolic parameter	White		Wheat		*P* ^1^		
	Pre	Post	Pre	Post	Time	Treatment	Interaction
BMI (kg/m^2^)	34.9 ± 0.9	34.7 ± 0.9	34.6 ± 0.9	34.4 ± 0.8	**< 0.0001**	0.16	0.89
SBP (mmHg)	122.7 ± 1.7	119.7 ± 1.8	119.8 ± 1.7	117.5 ± 1.7	**0.0087**	**0.03**	0.75
DBP (mmHg)	81.0 ± 1.4	79.1 ± 1.3	80.9 ± 1.0	77.8 ± 1.2	**0.0013**	0.38	0.41
Triglyceride (mg/dL)	112.9 ± 9.8	114.8 ± 9.7	112.8 ± 9.9	113.7 ± 8.9	0.68	0.73	0.33
Total cholesterol (mg/dL)	187.4 ± 5.3	180.8 ± 5.4	183.3 ± 5.8	180.7 ± 6.0	0.12	0.46	0.57
HDL-cholesterol (mg/dL)	39.8 ± 1.7	38.8 ± 1.8	40.3 ± 1.7	39.2 ± 1.5	0.20	0.54	0.25
LDL-cholesterol (mg/dL)	123.3 ± 5.3	119.1 ± 4.9	120.4 ± 5.3	116.7 ± 5.7	0.16	0.42	0.93
Serum CRP (mg/dL)	0.50 ± 0.1	0.53 ± 0.1	0.48 ± 0.1	0.47 ± 0.1	0.78	0.31	0.50
Serum IL-6 (pg/mL)	1.88 ± 0.2	1.94 ± 0.2	1.87 ± 0.1	1.88 ± 0.2	0.82	0.91	0.83
Serum TNF-α (pg/mL)	5.05 ± 0.5	5.63 ± 0.6	5.09 ± 0.5	6.39 ± 1.0	0.15	0.62	0.58
Bristol Stool Scores	3.87 ± 0.28	4.28 ± 0.26	4.50 ± 0.29	4.08 ± 0.22	0.98	0.37	0.03
Fecal Calprotectin (μg/g)^2^	32.5 ± 4.7	35.8 ± 5.2	43.5 ± 7.5	32.8 ± 4.6	0.47	0.48	0.52
Fecal MPO (ng/g)^2^	176 ± 24	157 ± 21	174 ± 11	116 ± 32	**0.003**	0.68	0.32
Fecal Total SCFA (μmol/g)	65.3 ± 7.3	68.3 ± 6.8	69.3 ± 6.8	68.2 ± 7.7	0.87	0.81	0.71
Fecal Straight-Chain SCFA (μmol/g)	61.6 ± 7.0	64.0 ± 6.4	65.2 ± 6.5	63.9 ± 7.3	0.92	0.82	0.74
Fecal Branched-Chain SCFA (μmol/g)	3.7 ± 0.5	4.3 ± 0.7	4.2 ± 0.5	4.3 ± 0.5	0.29	0.76	0.52
Fecal Acetic Acid (μmol/g)	31.0 ± 4.0	33.6 ± 3.6	32.5 ± 3.6	32.1 ± 3.6	0.72	0.99	0.63
Fecal Propionic Acid (μmol/g)	13.8 ± 1.6	15.1 ± 2.0	14.8 ± 1.6	14.5 ± 1.9	0.72	0.91	0.51
Fecal Butyric Acid (μmol/g)	13.6 ± 1.5	12.5 ± 1.4	14.2 ± 1.8	14.7 ± 2.1	0.82	0.49	0.59
Fecal Valeric Acid (μmol/g)	2.7 ± 0.3	2.4 ± 0.3	2.9 ± 0.3	2.4 ± 0.3	0.08	0.78	0.45
Fecal Hexanoic Acid (μmol/g)	0.5 ± 0.1	0.4 ± 0.1	0.8 ± 0.3	0.4 ± 0.1	0.11	0.93	0.92
Fecal Isobutyric Acid (μmol/g)	1.5 ± 0.2	1.8 ± 0.3	1.7 ± 0.2	1.7 ± 0.2	0.39	0.75	0.53
Fecal 2-Methylbutyratic Acid (μmol/g)	0.9 ± 0.1	1.1 ± 0.2	1.1 ± 0.1	1.1 ± 0.1	0.33	0.87	0.49
Fecal Isovaleric Acid (μmol/g)	1.2 ± 0.2	1.4 ± 0.2	1.4 ± 0.8	1.4 ± 0.2	0.92	0.53	0.83
Fecal 4-Methylvaleric Acid (μmol/g)	0.03 ± 0.01	0.03 ± 0.01	0.04 ± 0.01	0.09 ± 0.06	0.27	0.98	0.90

1 Data (means ± SEM, *n* = 39) were analyzed using repeated measures two-way ANOVA. BMI, body mass index; SBP, systolic blood pressure; DBP, diastolic blood pressure; HDL, high-density lipoprotein; LDL, low-density lipoprotein; CRP, C-reactive protein; IL-6, interleukin-6; TNF-a, tumor necrosis factor alpha; MPO, myeloperoxidase; SCFA, short-chain fatty acid.

2 One participant was excluded from fecal calprotectin and MPO analyses due to a technical error (*n* = 38). Values shown in bold are to highlight parameters that are statistically significant (*P* < 0.05).

### 3.4 Glucose tolerance

**Figure 3:**
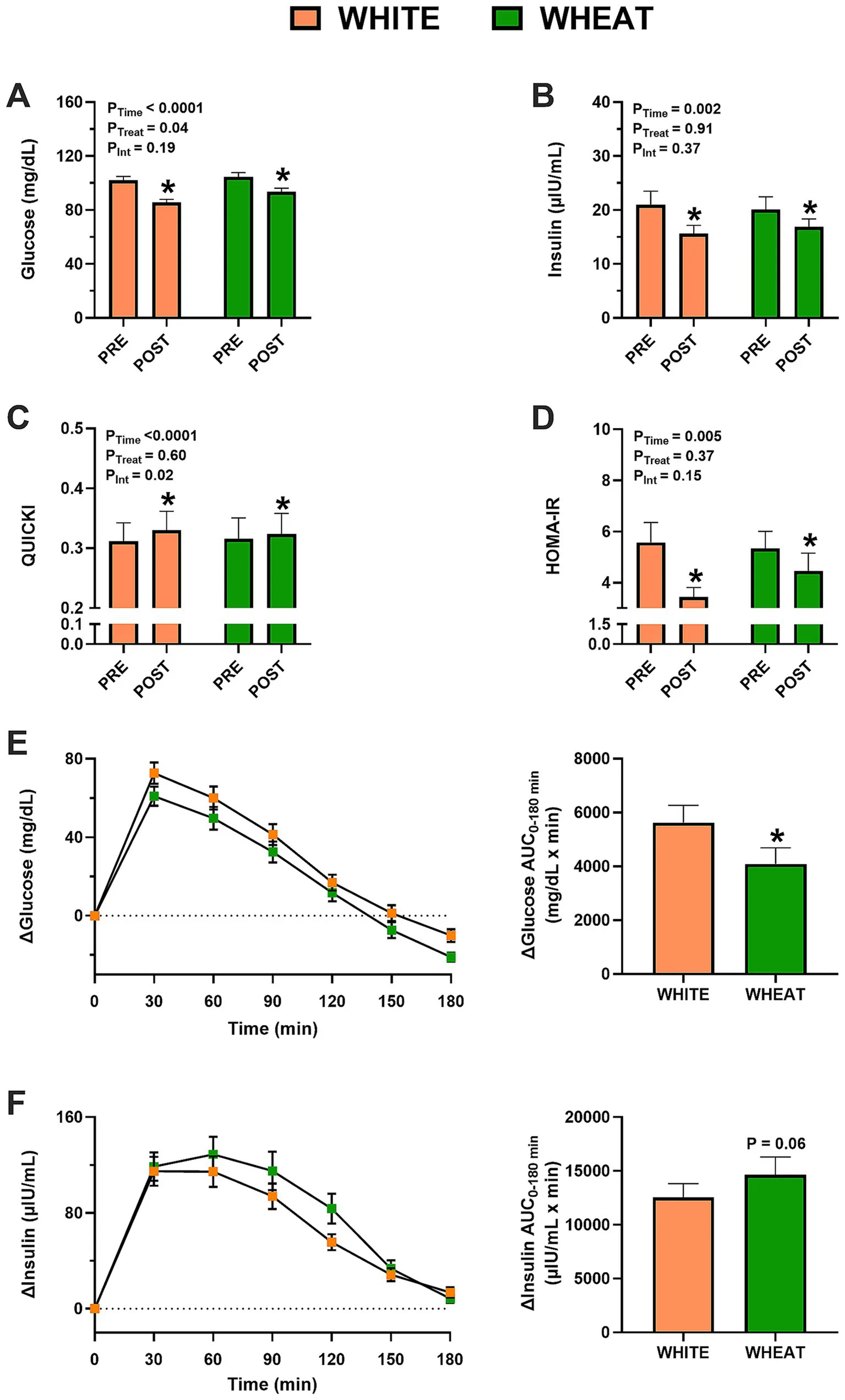
Fasting and postprandial plasma glucose and insulin concentrations, as well as calculated indices of insulin sensitivity and resistance, in adults with prediabetes consuming 160 g/day of whole wheat bread (WHEAT) or refined white bread (WHITE) as part of a fully controlled diet in a randomized, controlled crossover trial. Data (mean ± SEM; *n* = 39) were analyzed using two-way repeated-measures ANOVA to evaluate main effects of time and treatment, and their interaction. **(A)** Fasting plasma glucose was unaffected by treatment but decreased over time. **(B)** Fasting plasma insulin decreased over time, with no treatment or interaction effect. **(C)** Insulin sensitivity, estimated using the quantitative insulin sensitivity check index (QUICKI), increased over time during both study arms. **(D)** Insulin resistance, estimated using the homeostatic model assessment of insulin resistance (HOMA-IR), decreased over time during both study arms and was unaffected by treatment. **(E,F)** Postprandial glucose and insulin responses were assessed as area under the curve (AUC_0-180 min_) following an oral glucose tolerance test at the end of each intervention period, calculated from changes in biomarker concentrations relative to baseline (*t* = 0 min). Between-treatment differences in Δglucose and Δinsulin AUC_0-180 min_ were evaluated using paired *t*-tests. ΔGlucose AUC_0-180 min_ was significantly lower following WHEAT compared with WHITE, whereas Δinsulin AUC_0-180 min_ did not differ but tended to be higher during WHEAT (*p* = 0.06). Star indicates *p* < 0.05 compared with PRE.

We next evaluated the effect of WHEAT on glucose tolerance under controlled feeding conditions. No significant time × treatment interaction was observed for fasting glucose (*p* > 0.05). However, fasting glucose decreased by 13.7% over time (*p* < 0.0001; Figure 3A) independent of treatment. This time-dependent improvement occurred despite a 5.2% higher fasting glucose in WHEAT compared with WHITE at PRE (*p* = 0.04). Fasting insulin decreased by 4.3% over time (*p* = 0.002), with no treatment effect or interaction (Figure 3B). Consistent with these changes, HOMA-IR and QUICKI improved over time (*p* < 0.005) without treatment differences (Figures 3C,D). Regression analysis, controlling for within-subject repeated measures, indicated that fasting glucose was inversely correlated with QUICKI (r = −0.32; *p* = 0.045), whereas no association was observed with HOMA-IR at POST (*p* = 0.24; Supplementary Figure 1).

Postprandial responses during the OGTT differed by treatment. For each participant during each trial, AUC_0-180 min_ was calculated from the change in concentration relative to baseline (t = 0 min) using the trapezoidal method. Postprandial Δglucose AUC_0-180 min_ was 27% lower following WHEAT (4,092 ± 598 mg/dL x min) compared with WHITE (5,623 ± 645 mg/dL x min; p = 0.04; Figure 3E). In contrast, Δinsulin AUC_0-180 min_ did not differ between treatments, although a trend toward higher insulinemia during WHEAT was observed (*p* = 0.06; Figure 3F). BMI was not associated with fasting glucose or Dglucose AUC_0-180 min_ at POST (*p* = 0.35–0.43), suggesting that improved glucose tolerance was independent of the small but statistically significant changes in body mass (Supplementary Figure 1).

To further characterize glycemic responses, several OGTT-derived indices of glucose and insulin homeostasis were evaluated. No statistically significant differences were observed between treatments (*p* = 0.34–0.77; Supplementary Table 2) for whole-body insulin sensitivity (Matsuda index), early-phase insulin secretion (insulinogenic index), β-cell function (oral disposition index), or insulin secretion dynamics (Stumvoll index). However, the Stumvoll index was inversely correlated with Δglucose AUC_0-180 min_ (r = −0.35, *p* = 0.027; Supplementary Figure 1).

**Figure 4:**
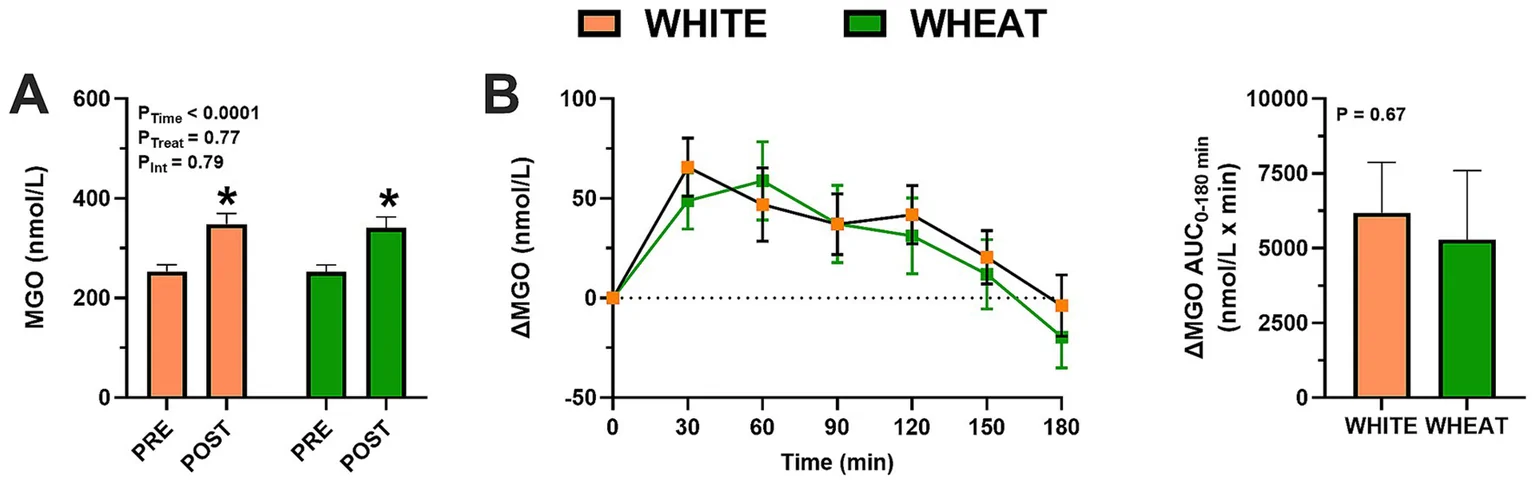
Fasting and postprandial plasma methylglyoxal (MGO) concentrations in adults with prediabetes consuming 160 g/day of whole wheat bread (WHEAT) or refined white bread (WHITE) as part of a fully controlled diet in a randomized, controlled crossover trial. Data are mean ± SEM (*n* = 39). **(A)** Two-way repeated-measures ANOVA was used to evaluate main effects of time and treatment, and their interaction. Fasting plasma MGO increased over time, with no treatment or interaction effect. **(B)** Postprandial MGO responses were assessed as area under the curve (AUC_0-180 min_) following an oral glucose tolerance test at the end of each intervention period, calculated from changes in concentration relative to baseline (t = 0 min). Between-treatment differences in ΔMGO AUC_0-180 min_ were evaluated using paired t-tests. ΔMGO AUC_0-180 min_ did not differ between WHEAT and WHITE. Star indicates *p* < 0.05 compared with PRE.

Plasma MGO was evaluated as a biomarker of glycation activity and carbonyl stress. A significant effect of time indicated that fasting MGO increased across both study arms (Figure 4A), with no treatment effect. Similarly, DMGO AUC_0-180 min_ did not differ between WHEAT and WHITE (Figure 4B). No associations were observed between MGO and indices of glucose or insulin homeostasis (Supplementary Figure 1).

### 3.5 Systemic inflammation

We next hypothesized that an anti-inflammatory activity in WHEAT would explain its observed glucose-lowering benefit. Participants’ serum CRP concentrations (Table 3) were within the range (0.3–1.0 mg/dL) of low-grade inflammation and being metabolically compromised (45). No significant effects of time, treatment, or interaction were observed for CRP, IL-6, or TNF-*α*. However, fasting glucose at POST was positively correlated with TNF-α (r = 0.36, *p* = 0.02; Supplementary Figure 1).

### 3.6 Intestinal health

Participants’ stool form and consistency characteristics, based on the Bristol Stool scale, were within the normal clinical range (46) across time and treatment (Table 3). A significant time × treatment interaction (*p* = 0.03) reflected minor baseline differences rather than treatment effects. Fecal calprotectin was unchanged (*p* > 0.05; Table 3), whereas fecal MPO decreased by 22% over time (*p* = 0.003), independent of treatment (*p* = 0.68) or time × treatment effects (*p* = 0.32). MPO and calprotectin were well-correlated (r = 0.50, *p* = 0.001; Supplementary Figure 1).

### 3.7 Gut microbiota diversity and composition

Diversity metrics were calculated using rarefied data to a sampling depth of 5,000 reads to account for uneven sampling depth between samples. Rarefaction curves plateaued near the selected rarefaction depth of 5,000 reads, reaching 95% of observed richness at a median sequencing depth of 4,700 reads, indicating that additional sequencing yielded relatively few new ASVs for most samples and supporting adequate coverage of microbial diversity. Two samples with sequencing depths below the threshold were excluded, resulting in a final sample size of *n* = 150. No statistically significant differences were observed between WHEAT and WHITE for any *α*-diversity metrics (Supplementary Figures 2A–C), including Observed Features (*p* = 0.97), Chao1 (*p* = 0.95), or Shannon diversity (*p* = 0.98). Similarly, *β*-diversity did not differ by treatment (Supplementary Figures 2D–E), as assessed by unweighted UniFrac (PERMANOVA, *p* = 0.71) and weighted UniFrac distances (PERMANOVA, *p* = 0.97).

After applying abundance and prevalence thresholds, feature tables yielded 6 phyla (Supplementary Figure 3A) and 40 genera (Supplementary Figure 3B) for differential abundance testing. Using a conservative statistical analysis with FDR correction, no treatment × time interactions or treatment main effects were detected at the phylum or genus level (Supplementary Table 3). Significant main effects of time were observed for a few taxa, including the genera *Streptococcus* (Q = 0.0004) and *Mediterraneibacter* (Q = 0.01), indicating modest temporal shifts in microbial composition independent of intervention.

**Figure 5:**
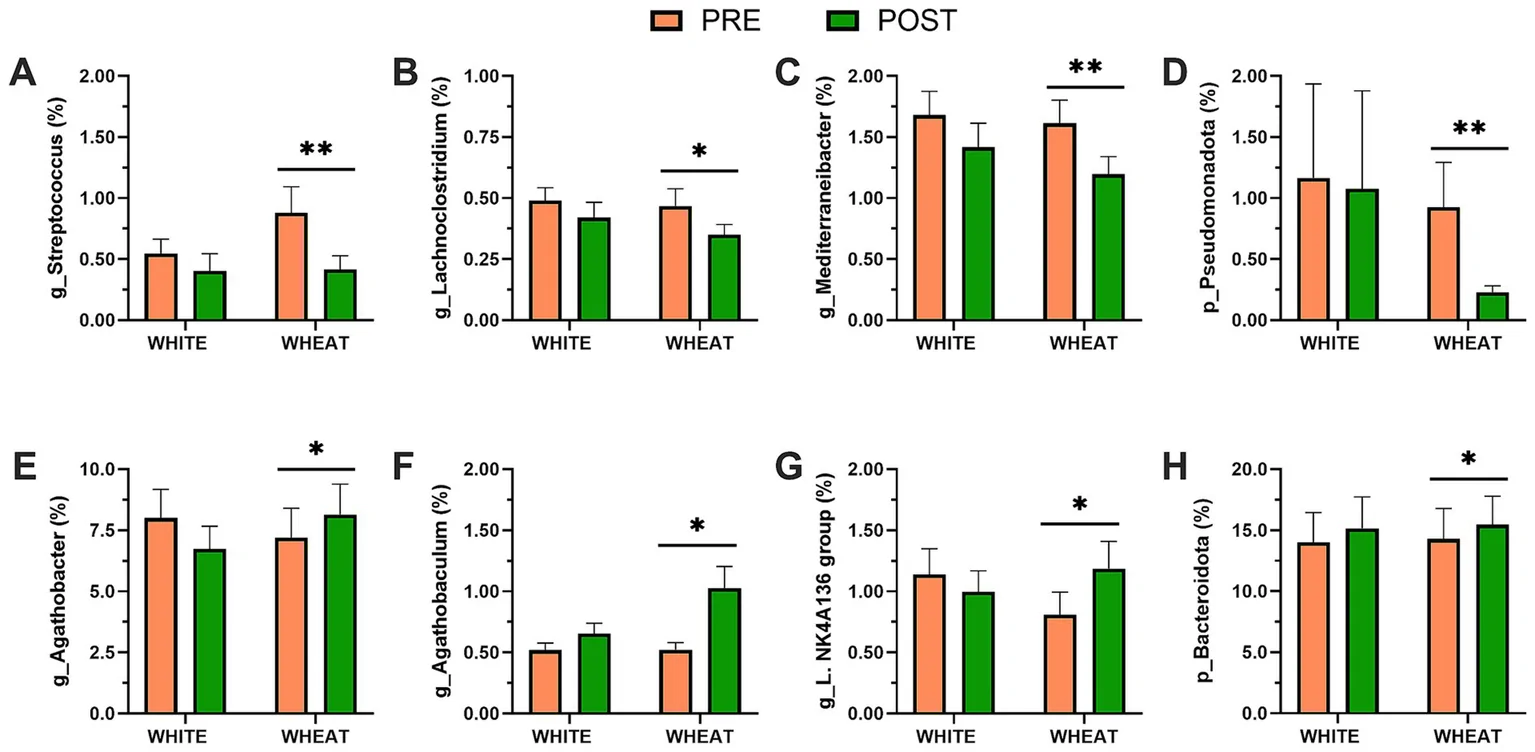
Relative abundance of selected gut microbial taxa from stool samples collected during the final 24 h of each 2-week intervention period in a randomized, controlled crossover trial. Participants consumed 160 g/day of whole wheat bread (WHEAT) or refined white bread (WHITE) as part of a fully controlled diet. Taxa shown were identified based on significant time, treatment, or time × treatment effects from linear mixed models using centered log-ratio-transformed data prior to false discovery rate correction. Significant taxa (*p* < 0.05) were back-transformed, and relative abundance values (%) are presented to facilitate interpretation of the magnitude and direction of microbial shifts between interventions. Decreased taxa during WHEAT include **(A)** *Streptococcus*; **(B)** *Lachnoclostridium*; **(C)** *Mediterraneibacter*, and **(D)** *Pseudomonadota*. Increased taxa during WHEAT include **(E)**, *Agathobacter*; F, *Agathobaculum*; **(G)** *Lachnospiraceae* NK4*A136 group*; and **(H)** *Bacteroidota*.

Because microbiota analyses were not powered to detect FDR-corrected effects, taxa-level results were further examined using a predefined exploratory framework with unadjusted *p*-values. Within this context, treatment-specific shifts in gut microbial composition were observed across the 14-day intervention periods (Supplementary Table 3), with within-treatment changes more pronounced during WHEAT compared with WHITE. *Agathobacter* and *Streptococcus* demonstrated nominally significant treatment × time interactions (*p* ≤ 0.05), suggesting divergent microbial responses between interventions. During WHEAT, participants exhibited decreases in multiple genera associated with inflammatory states (47–49), including *Streptococcus* (P_Interaction_ = 0.02), *Lachnoclostridium* (P_Time_ = 0.04), *Mediterraneibacter* (P_Time_ = 0.001), and members of the phylum *Pseudomonadota* (P_Time_ = 0.003; Figures 5A–D), whereas minimal change was observed during WHITE. Concurrently, several taxa commonly associated with saccharolytic fermentation and SCFA production (50–52) increased during WHEAT, including *Agathobacter* (P_Interaction_ = 0.03), *Agathobaculum* (P_Time_ = 0.01), *Lachnospiraceae NK4A136 group* (P_Time_ = 0.04), and members of the phylum *Bacteroidota* (P_Time_ = 0.05; Figures 5E–H). These changes were not observed during WHITE. Collectively, these patterns are consistent with a microbiota response to increased whole wheat–derived fiber within the controlled dietary context.

### 3.8 Microbial metabolites and predicted functions

Based on WHEAT influencing SCFA-producing taxa, we evaluated these microbial metabolites consistent with their known role in regulating glucose homeostasis. Contrary to our hypothesis, total SCFAs as well as total straight-chain and total branched-chain SCFAs exhibited no time, treatment, or interactive effects (Table 3). Individual SCFAs including acetic acid, propionic acid, butyric acid, valeric acid, hexanoic acid, isobutyric acid, 2-methylbutyric acid, isovaleric acid, and 4-methylvaleric acid also showed no significant main or interactive effects.

Because neither the controlled basal diet nor whole grain consumption appeared to influence these microbiota-derived metabolites, we used PICRUSt2 to infer the gut microbiome’s predicted functional capacity and identify potential pathways contributing to improved glucose tolerance. NSTI values varied across samples, with a subset exceeding 0.15, a commonly used threshold for increased uncertainty in functional inference; accordingly, PICRUSt2 results are interpreted as exploratory. The analysis yielded 6,048 KEGG Ortholog annotations, which were collapsed into 408 KEGG pathways. After filtering to identify ≥0.1% relative abundance in ≥10% of samples, 208 pathways were retained for downstream analyses.

**Figure 6:**
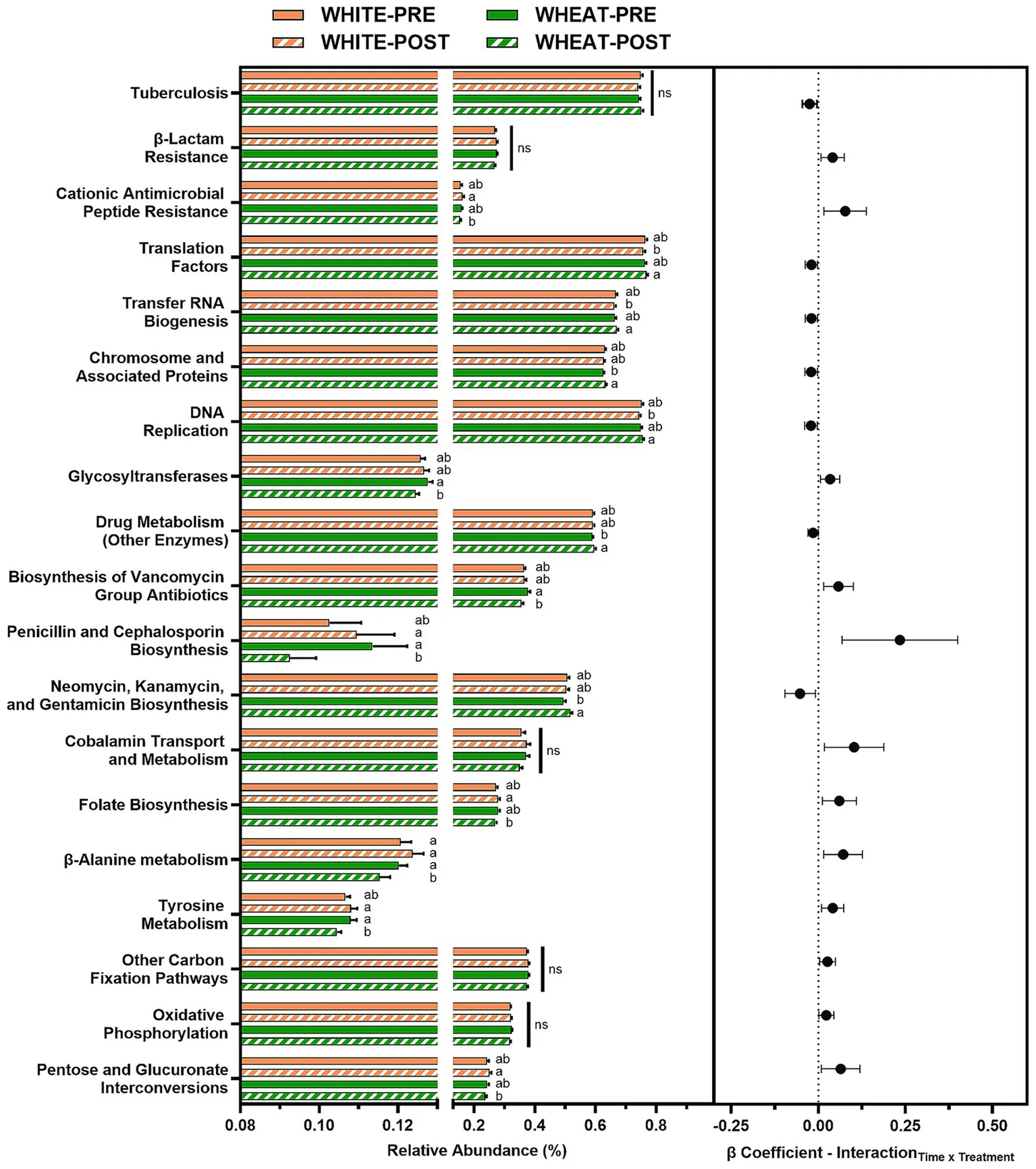
Predicted functional pathway abundances and interaction effects derived from KEGG annotations. Stool samples were collected for microbiota analysis during the final 24 h of each intervention arm in a randomized, controlled crossover trial in which participants (*n* = 39) consumed 160 g/day of whole wheat bread (WHEAT) or refined white bread (WHITE) as part of a fully controlled diet for 2 weeks. Microbial pathways shown were identified based on significant time × treatment interaction effects from linear mixed models using centered log-ratio (CLR)-transformed data prior to false discovery rate correction. (Left Panel) Data are back-transformed relative abundances (%) at PRE and POST for each intervention, with error bars representing SEM. Letters above bars denote *post hoc* comparisons performed on CLR-transformed data. Bars not sharing a common superscript are significantly different from each other (*p* < 0.05). (Right Panel) Forest plot of modeled interaction effect sizes (*β*; time × treatment), representing the differential longitudinal change between treatments. Positive interaction coefficients (*β* > 0) indicate pathways in which CLR abundance increased more (or decreased less) during WHITE relative to WHEAT, whereas negative coefficients (*β* < 0) indicate greater increases (or smaller decreases) during WHEAT relative to WHITE. Circles represent fixed-effect interaction coefficients (*β*), and error bars denote 95% confidence intervals. Coefficients are expressed in CLR units.

Several predicted microbial pathways exhibited time × treatment interactions prior to FDR correction (*p* < 0.05; Figure 6; Supplementary Table 4), including carbohydrate and energy metabolism (*n* = 3), amino acid and cofactor metabolism (*n* = 4), genetic information processing (*n* = 5), secondary metabolite biosynthesis and antimicrobial resistance (*n* = 4), and drug resistance and human disease (*n* = 3). Pentose and glucuronate interconversions and folate biosynthesis pathways were lower in WHEAT compared with WHITE at POST. Pathways of tyrosine metabolism and *β*-alanine metabolism showed lower enrichments in WHEAT compared to WHITE at POST while also exhibiting a within-WHEAT intervention decrease. Whereas, select pathways related to genetic information processing (DNA replication, translation factor, and tRNA biogenesis) were enriched in WHEAT. The glycosyltransferases pathway only showed a within-WHEAT decrease whereas the chromosome and associated proteins pathway showed a within-WHEAT increase. For secondary metabolite biosynthesis and antimicrobial resistance pathways, penicillin and cephalosporin biosynthesis showed a within-WHEAT decrease in enrichment in addition to being lower in WHEAT compared with WHITE at POST. Biosynthesis of vancomycin showed a within-WHEAT decrease, whereas drug metabolism (other enzymes) and biosynthesis of neomycin, kanamycin, and gentamicin exhibited a within-WHEAT increase. For drug resistance and human disease, only the cationic antimicrobial peptide resistance pathway showed lower enrichment in WHEAT compared with WHITE at POST.

### 3.9 Moderation of glycemic responses by gut microbiota

**Figure 7:**
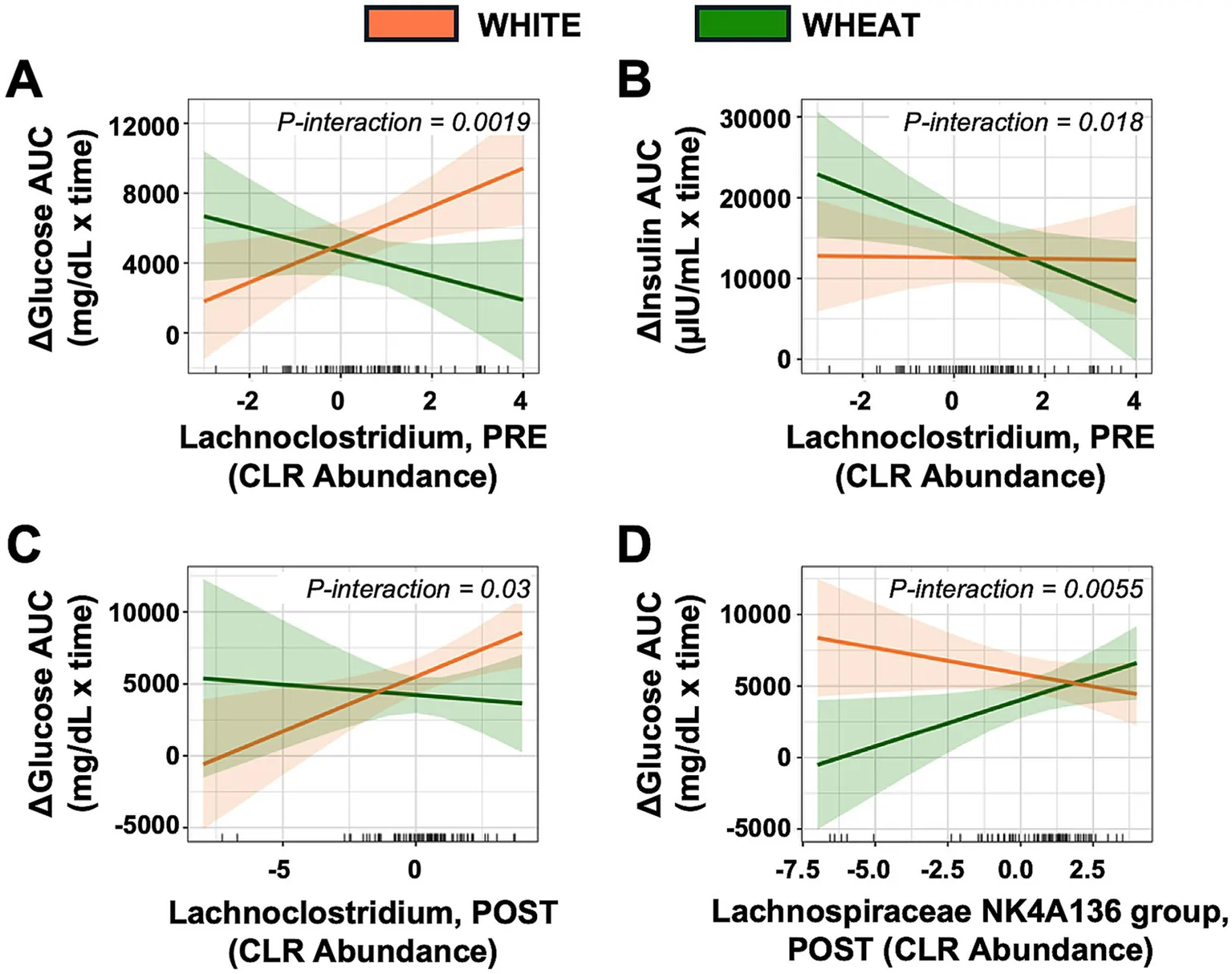
Baseline gut microbial abundance moderates glycemic responses to WHEAT in a randomized, controlled crossover trial in adults with prediabetes (*n* = 39). Data represent postprandial glucose (Δglucose AUC_0-180 min_) and insulin responses (Δinsulin AUC_0-180 min_) at POST, along with centered log-ratio (CLR)-transformed abundances of selected taxa measured at PRE or POST. Linear mixed models were used to evaluate treatment × abundance interactions. A significant treatment × abundance interaction indicates that the relationship between microbial abundance and glycemic response differed between WHEAT and WHITE. Divergent regression slopes suggest that microbial abundance modified the metabolic response to treatment, with steeper slopes indicating stronger associations between microbial abundance and glycemic response. Regression lines represent model-estimated values, with shading illustrating 95% confidence intervals for WHEAT (green) and WHITE (orange). Rug plots along the x-axis (tick marks for individual observations) indicate the distribution of CLR-transformed abundance values across participants. **(A)** *Lachnoclostridium* abundance at PRE significantly moderated Δglucose AUC_0-180 min_ and **(B)** Δinsulin AUC_0-180 min_, with higher baseline abundance associated with improved postprandial responses during WHEAT compared with WHITE, indicating a treatment-dependent relationship between microbial abundance and glycemic control. **(C)** *Lachnoclostridium* abundance at POST was associated with higher Δglucose AUC_0-180 min_ following WHITE but not WHEAT. **(D)** The *Lachnospiraceae NK4A136 group* at PRE also significantly moderated Δglucose AUC_0-180 min_, with higher baseline abundance associated with higher postprandial glucose responses during WHEAT.

We examined whether baseline gut microbial abundance moderated postprandial glucose tolerance responses under WHEAT versus WHITE using treatment × abundance interaction models in an exploratory analysis of genera that were responsive to WHEAT (Figures 5A–C,E). Because abundance at PRE reflects the host–microbe context entering each intervention period, moderation analyses used PRE abundance to evaluate whether baseline microbial differences influenced glycemic responses. Post-intervention abundance (POST) was analyzed separately to assess associations with glycemic outcomes following each diet. Supplementary Table 5 shows diet-specific associations of the six genera evaluated in moderation analysis. Two genera exhibited significant treatment-dependent associations with postprandial glycemic outcomes (Figure 7) and are further detailed below.

As shown in Figure 7A, baseline abundance of *Lachnoclostridium* significantly modified postprandial responses (P_Interaction_ = 0.0019). Higher PRE abundance was associated with lower Δglucose AUC_0-180 min_ under WHEAT but higher Δglucose AUC_0-180 min_ under WHITE (Figure 7A). A similar pattern was observed for Δinsulin AUC_0-180 min_ (P_Interaction_ = 0.02; Figure 7B), with higher PRE abundance associated with lower insulin responses during WHEAT and attenuated associations during WHITE. At POST, *Lachnoclostridium* abundance was not associated with Δglucose AUC_0-180 min_ under WHEAT but was positively associated with Δglucose AUC_0-180 min_ under WHITE (P_Interaction_ = 0.03; Figure 7C), with no associations observed for insulin. Baseline abundance of the *Lachnospiraceae NK4A136 group* also demonstrated treatment-dependent moderation (P_Interaction_ = 0.0055; Figure 7D). Higher PRE abundance was associated with higher Δglucose AUC_0-180 min_ under WHEAT and lower Δglucose AUC_0-180 min_ under WHITE. No associations were observed for Δinsulin AUC_0-180 min_ or for POST abundance. Together, these findings indicate that baseline gut microbial composition is associated with differential glycemic responses to whole wheat and refined white bread.

## 4 Discussion

This rigorously controlled RCT demonstrates that short-term whole wheat consumption improves postprandial glucose tolerance in adults with prediabetes, independent of changes in energy intake, body weight, insulinemia, carbonyl stress, inflammatory markers, or fecal SCFAs. These glycemic improvements were accompanied by coordinated shifts in gut microbial composition, including enrichment of carbohydrate-fermenting taxa (*Agathobacter*, *Agathobaculum*, *Lachnospiraceae NK4A136* group) (50–52) and depletion of taxa linked to less favorable metabolic phenotypes (*Streptococcus*, *Lachnoclostridium*, *Mediterraneibacter*) (47–49). Although no individual taxa reached FDR significance, the consistent pattern of directional changes supports a microbiota response to increased dietary substrates provided by whole wheat during WHEAT. Importantly, moderation analyses indicated that baseline gut microbial features were associated with interindividual variability in glycemic responsiveness. Collectively, these findings demonstrate that whole wheat improves postprandial glucose tolerance while exhibiting microbiome-associated variation in response, consistent with a role for gut microbial features in precision nutrition.

Current dietary guidelines recommend that at least half of grain intake be derived from whole grains (5), supported by evidence linking higher whole grain consumption with lower cardiometabolic risk (4). Meta-analyses of RCTs indicate that replacing refined grains with whole grains modestly improves glycemia, although effect sizes are smaller than those observed in prospective cohort studies (6). Notably, substantial interindividual variability in metabolic responses to whole grain intake is well reported (17), but the mechanisms underlying this heterogeneity remain incompletely understood. Differences in study populations, grain types, intervention duration, and limited dietary control likely contribute to this variability (53). By isolating whole wheat within a tightly controlled feeding design, our study extends prior work by simultaneously evaluating glycemic efficacy and variability in metabolic response in adults with prediabetes.

A key strength of this study is the rigorous dietary control, which enabled isolation of whole wheat–specific effects. Adherence was high (>98% of test bread consumed), with minimal intake of non-prescribed foods. Improvements in postprandial glucose occurred independent of adiposity, as body mass remained stable (<1% BMI change) across treatments. Diets were closely matched for energy and macronutrients, with only a small reduction in carbohydrates (~1%) and a planned increase in fiber during WHEAT (~7 g/day) compared with WHITE. Both diets exceeded typical U. S. fiber intakes (54), and differences in diet quality were driven solely by the test breads, allowing attribution of observed effects specifically to whole wheat.

Participants exhibited insulin resistance (HOMA-IR) at study inception, consistent with prediabetes (31). While both interventions reduced fasting glucose and insulin, only WHEAT improved postprandial glycemia. A trend toward higher postprandial insulinemia (*p* = 0.06) during WHEAT may reflect a compensatory response; however, this was not supported by improvements in OGTT-derived indices of insulin sensitivity or *β*-cell function. The absence of treatment effects on these indices, despite improved postprandial glycemia, suggests that mechanisms beyond conventional measures of insulin sensitivity or secretion contribute to the observed benefit. Blood pressure also improved similarly in both study arms, likely reflecting the overall higher quality of the controlled diets. In contrast, systemic inflammatory markers (CRP, IL-6, and TNF-*α*) and fecal calprotectin were unchanged, although fecal MPO decreased in both arms, suggesting reduced gut-level oxidative activity without altered neutrophil infiltration. These findings indicate that improvements in glucose tolerance during WHEAT occurred independently of measurable anti-inflammatory effects, consistent with prior short-term whole grain interventions (14), whereas longer-term studies may be required to detect anti-inflammatory benefits (55, 56).

Improvements in glycemia were also independent of carbonyl stress, as postprandial MGO responses did not differ between treatments. Unexpected increases in fasting MGO across both intervention arms suggest that factors beyond glycemia contributed to circulating MGO concentrations (19). MGO reflects the balance between production and detoxification and can arise from multiple sources, including environmental and dietary exposures, microbial metabolism, host carbohydrate, lipid, and amino acid metabolism, oxidative stress, and glyoxalase-mediated detoxification (19, 57). Consequently, changes in circulating MGO do not necessarily parallel changes in glucose homeostasis. One potential explanation is dietary exposure. Both intervention arms incorporated prescribed diets but lacked control for participants toasting baked grain products, which may have increased exposure to dicarbonyl compounds and Maillard reaction products generated during food processing and preparation (58). Microbial metabolism represents another possible source of MGO (57). However, our microbiome analyses did not identify significant changes in taxa commonly associated with MGO production (e.g., *Escherichia coli*), suggesting that alterations in microbial MGO generation are unlikely to explain the observed increase. Host metabolic regulation may also be a plausible contributor, although the present study did not evaluate pathways regulating MGO production or detoxification. In particular, future studies should examine both MGO-generating pathways and the glyoxalase system, which plays a central role in MGO clearance (19). Collectively, these findings indicate that short-term whole wheat consumption improves postprandial glucose tolerance independent of changes in carbonyl stress. Given the role of MGO in advanced glycation end-product formation and metabolic disease risk, future mechanistic studies should distinguish dietary, microbial, and host contributions to circulating MGO and determine whether alterations in glyoxalase-mediated detoxification contribute to changes in carbonyl stress during dietary interventions.

Because glycemic improvements were not explained by host metabolic or inflammatory changes, we evaluated microbial SCFAs as a potential mediator. Consistent with other short-term trials (59, 60), fecal SCFAs were unchanged despite improvements in postprandial glucose tolerance. In contrast, WHEAT increased the abundance of taxa associated with complex carbohydrate utilization and SCFA production, including *Agathobacter*, *Agathobaculum*, and *Lachnospiraceae NK4A136 group*, indicating microbial adaptation despite stable fecal SCFA concentrations. This apparent disconnect may partly reflect the fiber composition of whole wheat. Whole wheat contains structurally complex fiber fractions, including cellulose, hemicellulose, and arabinoxylans (11), and the increase in dietary fiber during WHEAT was driven primarily by insoluble fiber, whereas soluble fiber intake differed only modestly between interventions. Although these fiber fractions can support fermentation and SCFA production, they differ in fermentability and may preferentially enrich saccharolytic taxa capable of degrading complex polysaccharides through carbohydrate-active enzymes (CAZymes) (52, 61). Consequently, shifts in microbial composition may occur without detectable accumulation of SCFAs in feces. Interpretation of fecal SCFAs also requires caution because fecal concentrations reflect the balance among microbial production, host utilization, and absorption rather than total SCFA generation within the colon (62, 63). Most SCFAs produced during fermentation are rapidly consumed by colonocytes or absorbed into the circulation, and circulating rather than fecal SCFAs may more closely relate to glycemic outcomes (64). Thus, unchanged fecal SCFAs do not necessarily indicate that microbial fermentation or SCFA flux was unaffected by whole wheat consumption. Finally, because the present study was designed to evaluate whole wheat as a dietary exposure rather than individual fiber fractions, the observed microbial responses cannot be attributed to specific components of the wheat fiber matrix. Future studies incorporating purified or selectively enriched fibers, circulating SCFA measurements, and comprehensive metabolomics are needed to determine the relative contributions of arabinoxylans, cellulose, hemicellulose, and other whole wheat constituents to microbiome remodeling and metabolic outcomes.

WHEAT did not alter global microbial diversity, consistent with prior short-term interventions (65), but selectively enriched taxa capable of complex carbohydrate utilization, including *Agathobacter, Agathobaculum*, and *Lachnospiraceae NK4A136* group. Increased availability of fiber (e.g., arabinoxylans) and fiber-bound phytochemicals likely enhanced the ecological fitness of saccharolytic taxa adapted to structurally complex substrates (61), potentially promoting the production of bioactive metabolites beyond SCFAs that influence host glucose metabolism. For example, *Agathobacter* is increasingly linked to favorable glycemic control, with higher abundance associated with reduced risk of T2DM (66) and lower postprandial glucose excursions (67). Although butyrogenic (68), this was not reflected in fecal SCFAs in the present study. Instead, its bile salt hydrolase activity (69) suggests a potential mechanism involving microbial regulation of bile acid pools that can influence FXR- and TGR5-mediated signaling relevant to glucose metabolism. FXR- and TGR5 are bile acid receptors involved in the regulation of glucose homeostasis, insulin sensitivity, and energy metabolism (69). Although these pathways were not directly measured in the present study, they represent a plausible SCFA-independent pathway consistent with the observed microbial compositional changes and glycemic improvements. This hypothesis is consistent with prior evidence linking whole grain intake, increased *Agathobacter*, and improved glycemia (60), as well as whole grain–rich dietary patterns altering bile acid profiles in association with improved insulin resistance (70). WHEAT also enriched *Agathobaculum* and the *Lachnospiraceae NK4A136* group, taxa adapted to complex carbohydrate utilization, with prior evidence supporting substrate-specific selection by wheat-derived fibers (52). Although the metabolic role of *Lachnospiraceae* is context-dependent (51), its enrichment here likely reflects adaptation to the wheat matrix.

In parallel, WHEAT reduced the abundance of taxa associated with less favorable metabolic profiles, including *Streptococcus* and *Lachnoclostridium*. The latter encodes pathways for trimethylamine (TMA) production, which is subsequently biotransformed to trimethylamine oxide at the liver and linked to cardiometabolic disease risk (48, 71–73). Although the implications of TMAO remain debated (74), dietary patterns rich in whole grains are associated with lower TMAO and improved glycemia (75), suggesting that reductions in TMA-generating taxa may contribute to metabolic improvements. Consistent with this framework, *Lachnoclostridium* is inversely associated with diet quality and fiber-degrading taxa (76, 77), supporting competitive displacement during WHEAT, while suppression of *Streptococcus* aligns with evidence of antimicrobial activity by whole-grain substrates (78).

Predicted functional profiles provided additional, exploratory support for compositional restructuring of the microbiota by WHEAT, with shifts in pathways related to carbohydrate and energy metabolism, amino acid metabolism, and genetic information processing. Although based on inferred metagenomic content, the directional changes are consistent with a microbial community adapting to structurally complex carbohydrate substrates. In particular, lower enrichment of amino acid metabolism in WHEAT compared to WHITE may suggest decreased proteolytic fermentation and reduced production of detrimental metabolites such as TMA. Additionally, enrichment of pathways associated with core cellular processes during WHEAT may reflect enhanced metabolic activity or ecological competitiveness of saccharolytic taxa under whole wheat conditions, though caution is warranted when interpreting these inferences.

Moderation analyses revealed that baseline microbiome composition contributed to variability in glycemic response. *Lachnoclostridium* emerged as a significant diet-dependent moderator of postprandial glucose and insulin, with higher baseline abundance predicting poorer glucose tolerance under WHITE but improved responses under WHEAT. This adverse association persisted post-intervention under WHITE yet was attenuated following WHEAT. Notably, WHEAT decoupled this microbial signature from its potential negative metabolic impact and also reduced the relative abundance of *Lachnoclostridium* itself. Together, these findings suggest that individuals with high abundances of *Lachnoclostridium* may derive greater metabolic benefit from consuming whole grains compared with refined grains, consistent with prior cross-sectional studies linking *Lachnoclostridium* to diet quality and cardiometabolic risk (77). In contrast, *Lachnospiraceae NK4A136 group* exhibited opposing associations across diets, highlighting the context-dependent nature of microbiota–host metabolic interactions and suggesting limited capacity for further improvement among individuals with a more favorable microbial profile. Collectively, these findings suggest that whole wheat may mitigate unfavorable microbiome–glycemia relationships associated with refined-grain consumption. The convergence of compositional shifts and baseline-dependent moderation supports a model in which whole wheat reshapes the gut microbial ecosystem, while pre-existing microbial features influence the magnitude and direction of metabolic responses. Within this framework, the absence of changes in fecal SCFAs, despite compositional and functional changes, suggests that SCFA-independent pathways, including bile acid–mediated signaling (69), may contribute to the observed improvements in glucose tolerance and warrants future investigation.

This study has several strengths and limitations. A major strength is the rigorously controlled feeding crossover design, which enabled clear isolation of whole wheat–specific effects while achieving high adherence (>98%) and minimal changes in body weight. This level of control substantially reduces confounding from dietary heterogeneity and strengthens causal inference regarding the observed glycemic effects. Integration of detailed glycemic phenotyping with gut microbiota composition, predicted functional capacity, and moderation analyses also provides a precision nutrition framework for evaluating diet–microbe–host interactions and interindividual variability in glycemic response. The short-term intervention represents an intentional design feature that enabled assessment of early metabolic responses while minimizing confounding from long-term adaptations. Importantly, the present findings demonstrate that whole wheat can elicit measurable improvements in postprandial glucose tolerance within 2 weeks under tightly controlled feeding conditions. However, longer-duration studies will be necessary to determine whether these glycemic benefits persist over time, whether the observed microbial shifts stabilize, continue to evolve, or return toward baseline, and whether broader microbial metabolite profiles emerge as contributors to glycemic regulation. The modest sample size for microbiome analyses and reliance on predictive functional inference limit mechanistic resolution but support identification of biologically plausible signals under tightly controlled conditions. Similarly, the absence of comprehensive microbial metabolite profiling limits direct linkage of microbiome changes to host metabolic pathways. Accordingly, microbiome-related findings, including downstream moderation analyses, should be interpreted as exploratory yet hypothesis-generating, providing a foundation for multi-omics studies, including microbial and host metabolomics, to establish causal pathways and identify responder phenotypes.

In conclusion, whole wheat consumption under tight diet control significantly improved postprandial glucose tolerance in adults with prediabetes independently of body weight, inflammation, carbonyl stress, or fecal SCFAs. Despite minimal effects on global microbial diversity, whole wheat induced coordinated, directional changes in gut microbial composition and predicted functional capacity and modified baseline microbiome–glycemia relationships, indicating microbiome-associated variation in metabolic response rather than a uniform effect across individuals. These findings support whole wheat as an effective dietary strategy to improve postprandial glycemic control in individuals at elevated risk for T2DM and highlight substantial interindividual variability in response. Integrating glycemic outcomes with microbiome associations supports a precision nutrition framework in which gut microbial features help identify individuals most likely to benefit from whole grain interventions. Although mechanistic pathways cannot be definitively established, the combined absence of SCFA responses and presence of compositional, functional, and moderation signals suggests that whole wheat–induced metabolic effects may involve SCFA-independent microbial mechanisms that warrant further investigation. Future studies incorporating longer interventions, advanced metabolomic techniques, and responder-stratified designs will be critical to elucidate causal pathways, validate microbial predictors of response, and inform personalized dietary strategies to reduce progression from prediabetes to T2DM.

## Data Availability

The data presented in this study are not publicly available due to privacy and ethical restrictions. However, they are available on request from the corresponding author following institutional ethics approval. Requests to access these datasets should be directed to bruno.27@osu.edu.
