# Supplementary material for: Whole wheat bread improves postprandial glucose tolerance in adults with prediabetes: a controlled-feeding randomized crossover trial with microbiome-associated variation in response
Source: Front Nutr. 2026 Aug 28;13:1907908. doi: 10.3389/fnut.2026.1907908 (PMC13562025; doi:10.3389/fnut.2026.1907908)
Supplement: Supplementary file 1 [file Data_Sheet_1.PDF]

frontiers

Supplementary Material

Whole Wheat Bread Improves Postprandial Glucose Tolerance In Adults With Prediabetes: A Controlled-Feeding Randomized Crossover Trial with Microbiome-Associated Variation in Response

Jillian T Pierson,[1] Ariana H Bond,[1] Sisi Cao,[1] Shiqi Zhang,[1,2] Andrew Gold,[1,2] Huan Zhang,[1,2] Kaitlyn M Zamary,[1] Ayse Gurer,[1] Chao Guo,[1,2] Menuka Bhandari,[3] Matthew D. Teegarden,[4] Devin G Peterson,[5] Xiaokui Mo,[6] Jiangjiang Zhu,[1,2,*] Richard S. Bruno[1,*]

[1] Human Nutrition Program, The Ohio State University, Columbus, OH, USA, 43210

[2] James Comprehensive Cancer Center, The Ohio State University, Columbus, OH, USA, 43210

[3] College of Food, Agricultural, and Environmental Sciences Research and Analytical Services Core, The Ohio State University, Wooster, OH, USA, 44691

[4] Foods for Health Research Initiative, The Ohio State University, Columbus, OH, USA, 43210

[5] Department of Food Science and Technology, The Ohio State University, Columbus, OH, USA, 43210

[6] Center for Biostatistics, Department of Biomedical Informatics, The Ohio State University, Columbus, OH, USA, 43210

* co-corresponding authors:

Richard Bruno, PhD, RD, Carol S Kennedy Professor of Nutrition, Human Nutrition Program, The Ohio State University, Columbus, Ohio 43210; bruno.27@osu.edu or 614.292.5522

Jiangjiang Zhu, PhD, Associate Professor, Human Nutrition Program and James Comprehensive Cancer Center, The Ohio State University, Columbus, Ohio 43210; zhu.2484@osu.edu or 614.685.2226)

Supplementary Figure 1. Correlation matrix showing within-participant relationships between study variables measured at the end of each treatment arm during the randomized, controlled, crossover clinical trial in adults with prediabetes. Pairwise associations (n = 39 participants) between variables were assessed using linear regression accounting for repeated measures (rmcorr). The heatmap depicts the repeated-measures correlation (r_{rm}) for each pair. Cells marked with (*) indicate statistically significant ($P \leq 0.05$).

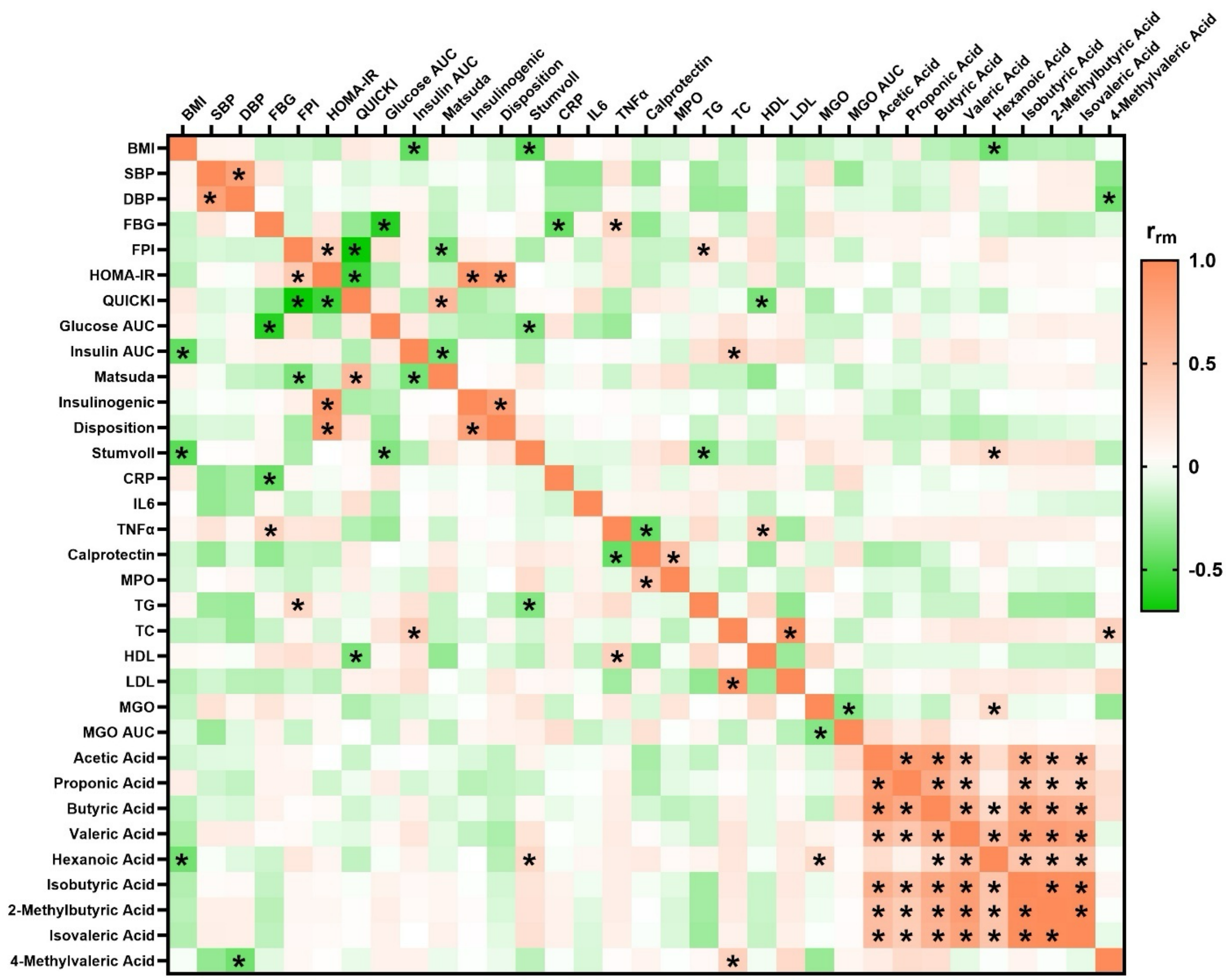

Supplementary Figure 2. α- and β-Diversity metrics at the end of each treatment arm during a randomized, controlled, crossover, clinical trial in persons with prediabetes. α-Diversity was performed to assess within-sample diversity at the end of each treatment was analyzed by observed features **(A)**, Chao1 **(B)**, and Shannon Index **(C)**, with no statistically significant differences between treatments observed ($P > 0.05$, Kruskal-Wallis test). β-Diversity was assessed using unweighted **(D)** and weighted **(E)** UniFrac distances and visualized by PCoA. No between-treatment significant differences were observed ($P > 0.05$; PERMANOVA).

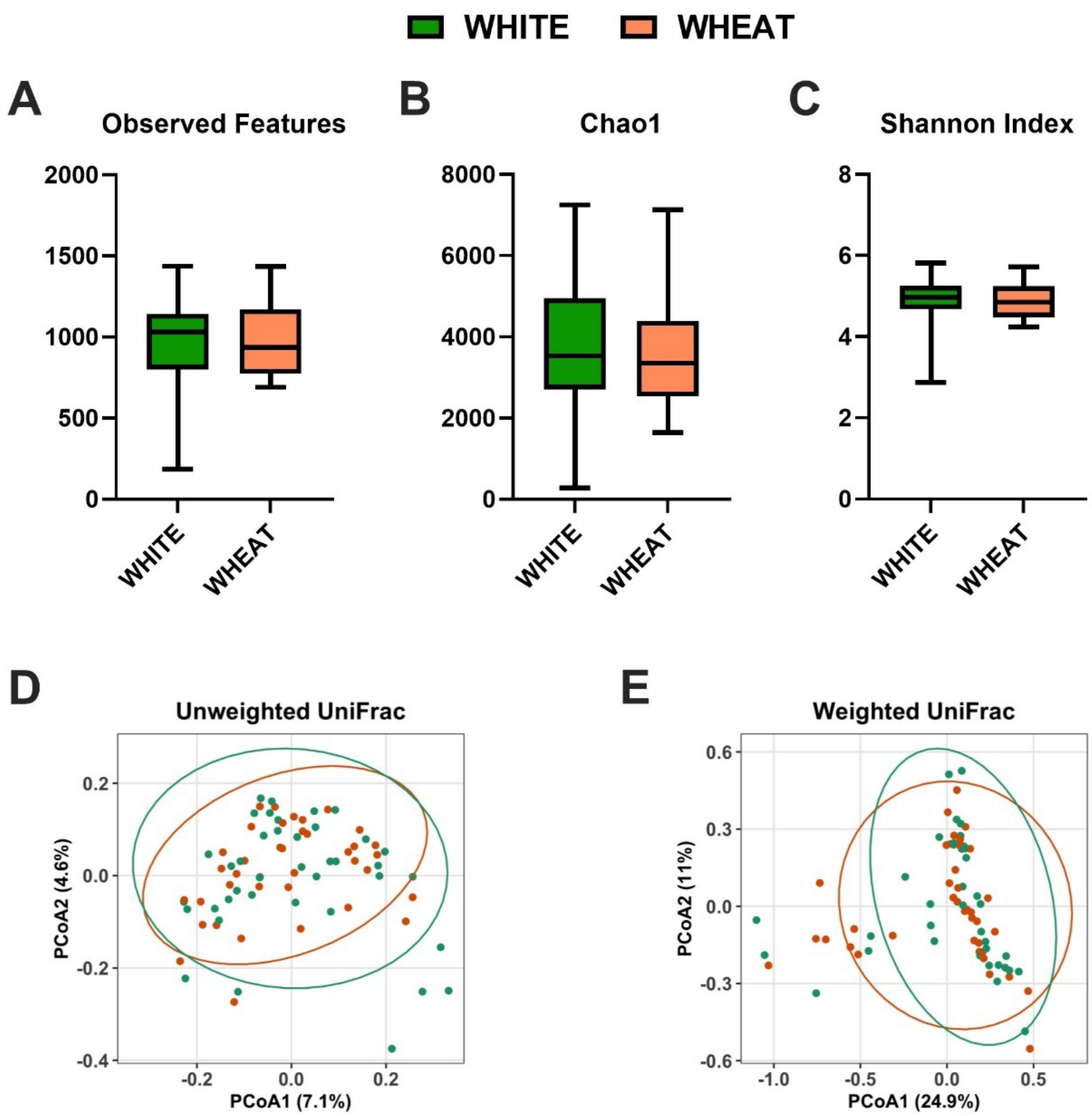

Supplementary Figure 3. Relative abundance (%) of gut microbiota at the phylum and genus levels from stool samples collected at PRE and POST of a randomized, controlled, crossover trial in 39 individuals with prediabetes consuming whole wheat or refined bread as part of fully controlled diet.

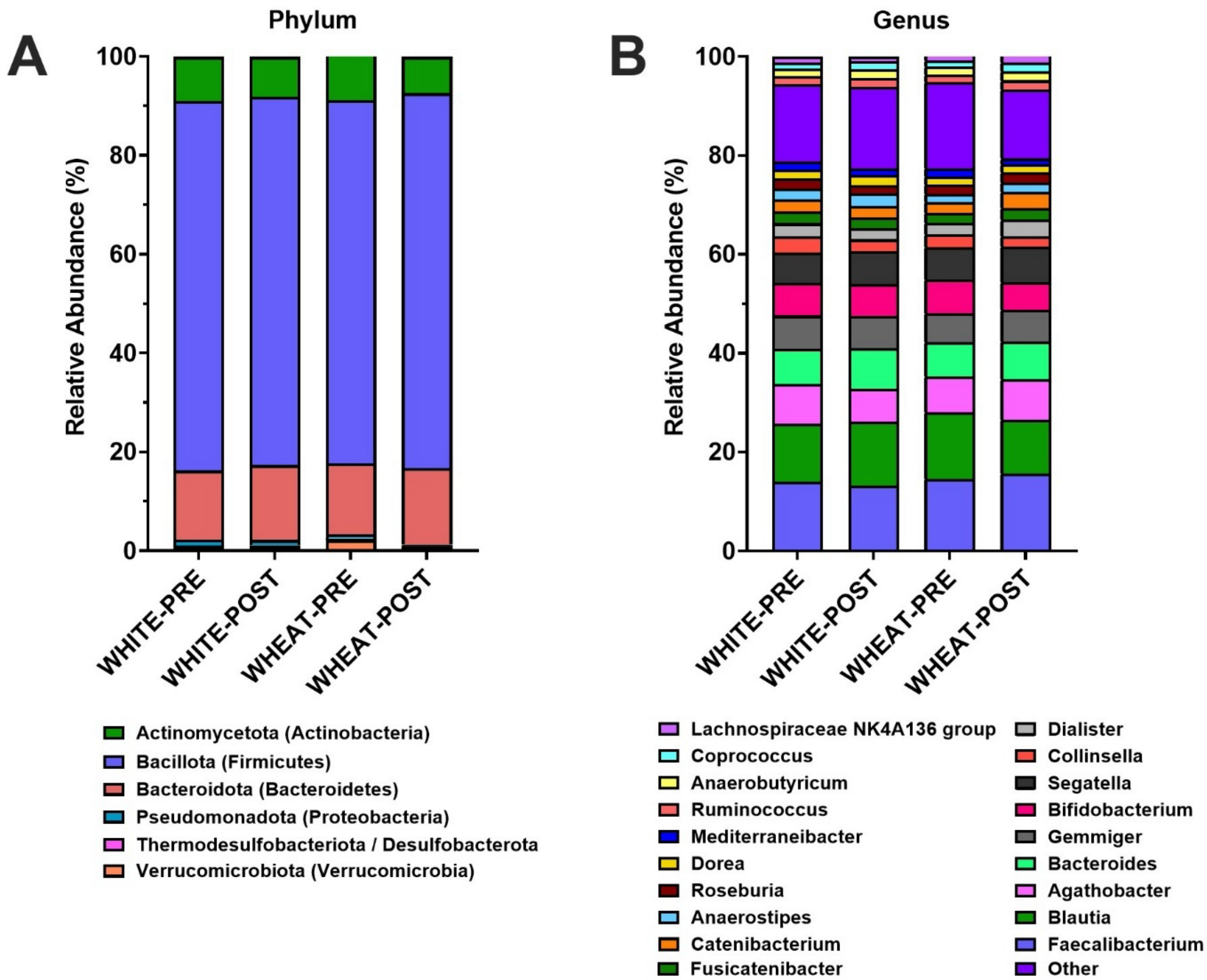

Supplementary Table 1. Prescribed diets were provided to participants enrolled in a two-arm randomized controlled trial to investigate the benefits of whole grains on cardiometabolic health. Participants were provided all foods based on a 4-day rotating menu during WHEAT and WHITE.

Day 1	Day 2	Day 3	Day 4
**BREAKFAST**	**BREAKFAST**	**BREAKFAST**	**BREAKFAST**
peanut butter and jelly sandwich:<br>2 slices test bread<br>peanut butter<br>jelly<br>Cocoa Krispie's<br>2% milk	Nutella sandwich:<br>2 slices test bread<br>Nutella<br>strawberries<br>sausage<br>orange juice	Eggo waffle<br>syrup<br>blueberries<br>scrambled eggs<br>hash browns	breakfast sandwich:<br>2 slices test bread<br>sausage<br>egg<br>cheese<br>butter<br>orange juice
**LUNCH**	**LUNCH**	**LUNCH**	**LUNCH**
turkey sandwich:<br>2 slices test bread<br>sliced turkey<br>cheddar cheese<br>tomato<br>lettuce<br>mayonnaise<br>potato chips	BBQ chicken sandwich:<br>2 slices test bread<br>BBQ chicken<br>coleslaw<br>Veggie Straws<br>apple	BLT club sandwich:<br>2 slices test bread<br>deli chicken<br>bacon<br>lettuce<br>tomato<br>mayonnaise	tuna sandwich:<br>2 slices test bread<br>canned tuna<br>mayonnaise
**DINNER**	**DINNER**	**DINNER**	**DINNER**
spaghetti<br>marinara sauce<br>meatballs<br>garlic bread stick	pork tenderloin<br>potatoes<br>carrots<br>gravy<br>butter	2 slices test bread<br>roast beef<br>mashed potatoes<br>gravy<br>corn	chicken enchiladas<br>Spanish-style rice<br>refried beans<br>salsa
**SNACK**	**SNACK**	**SNACK**	**SNACK**
trail mix<br>grapes	chocolate glazed donut<br>cucumber<br>ranch dressing	blueberry muffin<br>2% milk<br>kettle corn popcorn	chocolate chip cookie<br>Rice Krispie Treat<br>clementine<br>banana

Supplementary Table 2. Postprandial indices for insulin secretion, sensitivity, and β-cell function. Participants with prediabetes completed a two-arm, randomized crossover trial with 160 g/d of whole wheat or white bread in a controlled diet. After each two-week phase, participants underwent an oral glucose tolerance test, with blood samples collected every 30 minutes for 3 hours.

OGTT-Derived Index	Equation	WHITE	WHEAT	P[1]
Matsuda Index	$10{,}000 / \sqrt{(Glucose_{0} \times Insulin_{0} \times Mean\ Glucose_{0\text{-}180\ min} \times Mean\ Insulin_{0\text{-}180\ min})}$	4.04 ± 0.36	3.94 ± 0.52	0.77
Insulinogenic Index	$(Insulin_{30\ min - 0\ min}) / (Glucose_{30\ min - 0\ min})$	2.19 ± 0.34	2.27 ± 0.30	0.47
Oral Disposition Index	$\Delta Insulin_{0\ min - 30\ min} / \Delta Glucose_{0\ min - 30\ min} \times 1 / Insulin_{0\ min}$	7.64 ± 1.32	8.28 ± 2.20	0.46
Stumvoll Index	$0.222 - 0.00333 \times BMI - 0.0000779 \times Insulin_{120\ min} - 0.000422 \times Age$	0.084 ± 0.003	0.083 ± 0.003	0.34

[1] Data (means ± SEM, n = 39) were analyzed using a two-tailed, paired Student's t-test.

Supplementary Table 3. Differential abundance of gut microbiota at the phylum and genus levels from stool samples collected during a randomized, controlled, clinical trial in 39 individuals with prediabetes who consumed 160 g/d of wheat bread or white bread as part of a fully controlled diet.[1,2]

Taxa (Phylum or Genus)	WHITE-PRE	WHITE-POST	WHEAT-PRE	WHEAT-POST	P_{Time}	P_{Treat}	P_{Int}
Actinomycetota (Actinobacteria)	9.0 ± 1.7	8.2 ± 1.5	8.8 ± 2.1	7.4 ± 1.4	0.94	0.93	0.45
Bifidobacterium	6.6 ± 1.7	6.4 ± 1.7	6.8 ± 2.1	5.6 ± 1.4	0.32	0.36	0.71
Collinsella	3.2 ± 0.9	2.4 ± 0.4	2.6 ± 0.6	2.1 ± 0.4	0.53	0.36	0.78
Bacillota (Firmicutes)	74.7 ± 2.6	74.5 ± 2.4	73.5 ± 2.8	75.9 ± 2.3	0.65	0.32	0.83
Acutalibacter	1.1 ± 0.2	0.8 ± 0.2	0.8 ± 0.2	0.6 ± 0.1	0.13	0.45	0.57
Agathobacter	8.0 ± 1.2	6.7 ± 0.9	7.2 ± 1.2	8.1 ± 1.2	**0.02**	0.07	**0.03**
Agathobaculum	0.5 ± 0.1	0.7 ± 0.1	0.5 ± 0.1	1.0 ± 0.2	**0.01**	0.96	0.09
Anaerobutyricum	1.6 ± 0.2	1.8 ± 0.3	1.6 ± 0.2	1.8 ± 0.2	0.74	0.45	0.22
Anaerostipes	2.2 ± 0.4	2.7 ± 0.4	1.7 ± 0.3	1.9 ± 0.4	0.69	0.36	0.08
Blautia	11.7 ± 1.5	12.9 ± 1.7	13.5 ± 2.0	11.0 ± 1.1	0.22	0.14	0.08
Catenibacterium	2.4 ± 1.0	2.3 ± 0.9	2.2 ± 0.8	3.4 ± 1.2	0.57	0.39	0.52
Christensenellaceae R-7 group	0.8 ± 0.2	0.7 ± 0.2	0.7 ± 0.2	1.0 ± 0.3	0.54	0.82	0.46
Coprococcus	1.3 ± 0.2	1.5 ± 0.4	1.2 ± 0.2	1.8 ± 0.5	0.13	0.65	0.65
Dialister	2.6 ± 0.6	2.3 ± 0.6	2.3 ± 0.6	3.3 ± 0.9	0.53	0.28	0.08
Dorea	1.8 ± 0.2	2.0 ± 0.2	1.6 ± 0.2	1.6 ± 0.2	0.86	0.91	0.25
Enterocloster	0.8 ± 0.2	0.6 ± 0.1	0.6 ± 0.1	0.5 ± 0.1	0.06	0.46	0.25
Erysipelotrichaceae UCG-003	0.6 ± 0.1	0.9 ± 0.3	0.7 ± 0.1	0.8 ± 0.2	0.36	0.96	0.94
Faecalibacterium	14.1 ± 1.7	13.2 ± 1.6	14.6 ± 1.8	15.6 ± 1.5	0.10	0.81	0.21
Fusicatenibacter	2.4 ± 0.4	2.2 ± 0.5	2.0 ± 0.2	2.4 ± 0.3	0.82	0.75	0.46
Gemmiger	6.8 ± 1.0	6.5 ± 1.0	5.9 ± 1.0	6.3 ± 0.9	0.17	0.45	0.42
Holdemanella	1.1 ± 0.4	1.1 ± 0.6	1.1 ± 0.4	1.3 ± 0.6	0.87	0.35	0.25
Intestinibacter	0.2 ± 0.1	0.3 ± 0.1	0.2 ± 0.1	0.5 ± 0.3	0.37	0.78	0.68
Lachnoclostridium	0.5 ± 0.1	0.4 ± 0.1	0.5 ± 0.1	0.4 ± 0.0	**0.04**	0.66	0.75
Lachnospiraceae NC2004 group	0.8 ± 0.1	0.8 ± 0.1	0.7 ± 0.1	0.8 ± 0.1	0.08	0.52	0.18
Lachnospiraceae NK4A136 group	1.1 ± 0.2	1.0 ± 0.2	0.8 ± 0.2	1.2 ± 0.2	**0.04**	0.49	0.14
Mediterraneibacter	1.7 ± 0.2	1.4 ± 0.2	1.6 ± 0.2	1.2 ± 0.1	**0.001**	0.53	0.07
Megamonas	1.1 ± 0.9	1.1 ± 0.8	0.7 ± 0.6	0.0 ± 0.0	0.60	**0.05**	0.63
Megasphaera	0.5 ± 0.2	0.5 ± 0.2	1.0 ± 0.4	0.5 ± 0.2	0.09	0.20	0.13

Monoglobus	0.7 ± 0.1	0.5 ± 0.1	0.7 ± 0.1	0.6 ± 0.1	0.36	0.15	0.50
Phascolarctobacterium	0.8 ± 0.2	0.4 ± 0.1	0.7 ± 0.2	0.5 ± 0.1	0.16	0.73	0.90
Romboutsia	0.7 ± 0.1	1.9 ± 0.7	0.9 ± 0.3	0.5 ± 0.1	0.46	0.08	0.80
Roseburia	2.0 ± 0.4	1.6 ± 0.2	1.9 ± 0.3	2.0 ± 0.4	0.44	0.64	0.25
Ruminococcus	1.6 ± 0.3	1.7 ± 0.3	1.6 ± 0.3	1.9 ± 0.4	0.27	0.12	0.16
Streptococcus	0.5 ± 0.1	0.4 ± 0.1	0.9 ± 0.2	0.4 ± 0.1	**< 0.001**	0.09	**0.02**
Ruminococcaceae UCG-002	0.9 ± 0.2	1.1 ± 0.2	0.9 ± 0.3	1.4 ± 0.3	0.47	0.19	0.20
Ruminococcus gauvreauii group	0.6 ± 0.1	0.7 ± 0.1	0.6 ± 0.1	0.5 ± 0.1	0.91	0.33	0.69
Bacteroidota (Bacteroidetes)	14.0 ± 2.5	15.2 ± 2.6	14.3 ± 2.5	15.5 ± 2.3	**0.05**	0.73	0.11
Alistipes	0.9 ± 0.2	1.0 ± 0.2	1.2 ± 0.3	0.9 ± 0.2	0.61	0.95	0.38
Bacteroides	7.1 ± 1.4	8.2 ± 1.7	7.0 ± 1.4	7.7 ± 1.6	0.60	0.69	0.61
Parabacteroides	0.7 ± 0.2	0.6 ± 0.1	0.6 ± 0.1	0.5 ± 0.1	0.73	0.96	0.28
Segatella	6.2 ± 2.5	6.6 ± 2.6	6.6 ± 2.5	7.3 ± 2.4	0.23	0.58	0.72
Pseudomonadota (Proteobacteria)	1.2 ± 0.8	1.1 ± 0.8	0.9 ± 0.4	0.2 ± 0.1	**0.003**	0.40	0.10
Escherichia-Shigella	0.9 ± 0.8	0.9 ± 0.9	0.7 ± 0.4	0.1 ± 0.1	0.17	0.19	0.85
Verrucomicrobiota (Verrucomicrobia)	0.9 ± 0.3	0.9 ± 0.5	2.3 ± 1.4	0.9 ± 0.4	0.61	0.94	0.42
Akkermansia	1.0 ± 0.4	1.0 ± 0.6	2.8 ± 1.6	1.0 ± 0.4	0.78	0.96	0.72

[1] Relative abundance of taxa (means ± SEM) at PRE and POST of each treatment arm.

[2] Data were transformed using centered-log-ratio (CLR) and analyzed using linear mixed modeling with time, treatment, and their interaction as fixed effects and participants as a random effect. After applying false discovery rate (FDR) correction using the Benjamini-Hochberg method, only two taxa remained significant for time effect only: Mediterraneibacter ($Q_{Time} = 0.01$) and Streptococcus ($Q_{Time} = 0.0004$). There were no significant treatment or interaction effects (time x treatment) after FDR correction ($Q > 0.1$) for all other taxa (data not shown).

Supplementary Table 4. Predicted gene-enrichment of the gut microbiota community using KEGG pathways from stool samples collected during a randomized, controlled, clinical trial in 39 individuals with prediabetes investigating the benefits of whole wheat compared to refined wheat on glycemia**.** Pathways were predicted using PiCrust2, in which KEGG orthologs were collapsed into KEGG pathways. [1,2]

Classification	IDs	KEGG Pathway	WHITE-PRE	WHITE-POST	WHEAT-PRE	WHEAT-POST	P_{Time}	P_{Treat}	P_{Int}
Carbohydrate & Energy Metabolism	ko00040	Pentose and Glucuronate Interconversions	0.242 ± 0.006	0.251 ± 0.006	0.243 ± 0.005	0.236 ± 0.005	0.18	0.78	**0.03**
	ko00650	Butanoate Metabolism	0.224 ± 0.002	0.226 ± 0.002	0.223 ± 0.002	0.221 ± 0.001	0.36	0.87	0.10
	ko00660	C_5-Branched Dibasic Acid Metabolism	0.422 ± 0.004	0.418 ± 0.004	0.425 ± 0.005	0.412 ± 0.004	0.03	0.64	0.32
	ko00190	Oxidative Phosphorylation	0.319 ± 0.002	0.322 ± 0.002	0.324 ± 0.002	0.319 ± 0.002	0.07	0.07	**0.04**
	ko00720	Other Carbon Fixation Pathways	0.373 ± 0.003	0.379 ± 0.003	0.378 ± 0.003	0.374 ± 0.003	0.20	0.11	**0.02**
Glycan Metabolism	ko00510	N-Glycan Biosynthesis	0.148 ± 0.008	0.155 ± 0.009	0.161 ± 0.009	0.144 ± 0.006	0.07	0.10	0.07
	ko00511	Other Glycan Degradation	0.400 ± 0.025	0.433 ± 0.032	0.430 ± 0.026	0.420 ± 0.030	0.44	0.10	0.17
Lipid Metabolism	ko00074	Mycolic Acid Biosynthesis	0.514 ± 0.006	0.505 ± 0.005	0.509 ± 0.005	0.512 ± 0.006	0.45	0.29	0.07
Amino Acid Metabolism	ko00340	Histidine Metabolism	0.484 ± 0.004	0.479 ± 0.005	0.478 ± 0.004	0.483 ± 0.005	0.24	0.19	0.08
	ko00410	β-Alanine Metabolism	0.121 ± 0.003	0.124 ± 0.003	0.120 ± 0.002	0.115 ± 0.003	**0.03**	0.98	**0.01**
	ko00310	Lysine Degradation	0.123 ± 0.003	0.127 ± 0.003	0.124 ± 0.003	0.121 ± 0.003	0.28	0.80	0.06
	ko00350	Tyrosine Metabolism	0.107 ± 0.001	0.108 ± 0.002	0.108 ± 0.002	0.105 ± 0.001	**0.01**	0.38	**0.01**
	ko00330	Arginine and Proline Metabolism	0.181 ± 0.002	0.183 ± 0.002	0.182 ± 0.001	0.178 ± 0.001	0.06	0.64	**0.05**
Nucleotide Metabolism	ko00240	Pyrimidine Metabolism	0.540 ± 0.003	0.538 ± 0.004	0.537 ± 0.003	0.542 ± 0.003	0.10	0.31	0.08
Biosynthesis of Secondary Metabolites	ko00311	Penicillin and Cephalosporin Biosynthesis	0.103 ± 0.008	0.110 ± 0.010	0.114 ± 0.009	0.093 ± 0.007	**0.00**	0.09	**0.01**
	ko01055	Biosynthesis of Vancomycin Group Antibiotics	0.364 ± 0.005	0.365 ± 0.006	0.377 ± 0.007	0.356 ± 0.006	**0.00**	**0.03**	**0.01**
	ko00332	Carbapenem Biosynthesis	0.674 ± 0.005	0.663 ± 0.006	0.670 ± 0.006	0.674 ± 0.005	0.50	0.47	0.08
	ko00524	Neomycin, Kanamycin, Gentamicin Biosynthesis	0.507 ± 0.007	0.505 ± 0.008	0.495 ± 0.008	0.516 ± 0.007	**0.01**	0.10	**0.02**
	ko00525	Acarbose and Validamycin Biosynthesis	0.685 ± 0.009	0.685 ± 0.009	0.702 ± 0.010	0.679 ± 0.010	**0.02**	0.09	0.11
	ko00966	Glucosinolate Biosynthesis	0.747 ± 0.006	0.743 ± 0.007	0.754 ± 0.008	0.735 ± 0.005	0.08	0.54	0.33

Xenobiotics Biodegradation	ko00983	Drug Metabolism - Other Enzymes	0.592 ± 0.003	0.592 ± 0.004	0.589 ± 0.004	0.597 ± 0.004	**0.01**	0.29	0.05
Metabolism of Cofactors and Vitamins	ko00790	Folate Biosynthesis	0.272 ± 0.005	0.279 ± 0.005	0.279 ± 0.005	0.269 ± 0.005	0.06	0.17	**0.02**
	ko00780	Biotin Metabolism	0.593 ± 0.008	0.601 ± 0.007	0.600 ± 0.008	0.590 ± 0.006	0.18	0.28	0.07
Genetic Information Processing	ko00970	Aminoacyl-tRNA Biosynthesis	0.742 ± 0.006	0.733 ± 0.006	0.737 ± 0.006	0.742 ± 0.006	0.41	0.41	0.08
	ko03009	Ribosome Biogenesis	0.709 ± 0.005	0.703 ± 0.006	0.704 ± 0.005	0.713 ± 0.006	0.14	0.41	0.08
	ko03010	Ribosome Translation	0.710 ± 0.005	0.702 ± 0.006	0.707 ± 0.005	0.711 ± 0.005	0.41	0.55	0.08
	ko3011	Ribosome	0.710 ± 0.005	0.702 ± 0.006	0.707 ± 0.005	0.711 ± 0.005	0.41	0.55	0.08
	ko03012	Translation Factors	0.765 ± 0.005	0.758 ± 0.006	0.762 ± 0.005	0.768 ± 0.005	0.17	0.49	**0.04**
	ko03016	Transfer RNA Biogenesis	0.667 ± 0.004	0.662 ± 0.005	0.663 ± 0.004	0.670 ± 0.004	0.07	0.30	**0.03**
	ko03019	Messenger RNA Biogenesis	0.670 ± 0.003	0.664 ± 0.003	0.668 ± 0.003	0.673 ± 0.003	0.29	0.60	0.09
	ko03029	Mitochondrial Biogenesis	0.814 ± 0.005	0.805 ± 0.006	0.810 ± 0.005	0.817 ± 0.005	0.28	0.54	0.07
	ko03030	DNA Replication	0.752 ± 0.005	0.745 ± 0.005	0.748 ± 0.004	0.756 ± 0.004	0.12	0.43	0.02
	ko03032	DNA Replication Proteins	0.850 ± 0.004	0.843 ± 0.005	0.844 ± 0.005	0.853 ± 0.003	0.07	0.25	**0.03**
	ko03036	Chromosome and Associated Proteins	0.631 ± 0.003	0.627 ± 0.003	0.625 ± 0.004	0.633 ± 0.003	**0.04**	0.16	**0.03**
	ko03400	DNA Repair and Recombination Proteins	0.723 ± 0.004	0.717 ± 0.004	0.719 ± 0.004	0.725 ± 0.004	0.17	0.43	0.06
	ko03430	Mismatch Repair	0.812 ± 0.004	0.805 ± 0.005	0.809 ± 0.004	0.815 ± 0.004	0.28	0.57	0.10
	ko03440	Homologous Recombination	0.797 ± 0.005	0.790 ± 0.005	0.793 ± 0.004	0.800 ± 0.004	0.19	0.43	0.05
	ko01003	Glycosyltransferases	0.126 ± 0.001	0.127 ± 0.001	0.128 ± 0.001	0.124 ± 0.001	**0.01**	0.10	**0.02**
Drug Resistance	ko01503	Cationic Antimicrobial Peptide Resistance	0.155 ± 0.004	0.161 ± 0.004	0.158 ± 0.004	0.153 ± 0.003	0.13	0.24	**0.02**
	ko01501	β-Lactam Resistance	0.269 ± 0.003	0.274 ± 0.003	0.274 ± 0.003	0.268 ± 0.003	0.06	0.12	**0.02**
Organismal Systems	ko04980	Cobalamin Transport and Metabolism	0.356 ± 0.012	0.373 ± 0.011	0.371 ± 0.011	0.350 ± 0.008	0.09	0.15	**0.02**
	ko04910	Insulin Signaling Pathway	0.467 ± 0.006	0.462 ± 0.005	0.455 ± 0.007	0.467 ± 0.006	0.11	0.09	0.11
	ko04975	Fat Digestion and Absorption	0.382 ± 0.012	0.374 ± 0.013	0.371 ± 0.016	0.388 ± 0.014	0.09	0.17	0.11
Human Diseases	ko05152	Tuberculosis	0.750 ± 0.006	0.741 ± 0.006	0.742 ± 0.006	0.752 ± 0.006	0.10	0.21	**0.02**

frontiers

Supplementary Table 5. Diet-specific associations between selected taxa abundance (PRE or POST) and postprandial outcomes, and their moderation by treatment from linear mixed-effects models.[1]

	**ΔGlucose AUC$_{0\text{-}180\ min}$**					**ΔInsulin AUC$_{0\text{-}180\ min}$**				
**PRE Abundance**	**β_{WHEAT}**	**β_{WHITE}**	**β_{Int}**	**CI_{Int}**	**P_{Int}**	**β_{WHEAT}**	**β_{WHITE}**	**β_{Int}**	**CI_{Int}**	**P_{Int}**
Streptococcus	-303.5	-108.6	-194.8	-1256, 866	0.72	-1509.1	155.5	-1664.6	-3323, -6	0.06
Lachnoclostridium	-685.8	1089.9	-1775.8	-2819, -733	**0.0019**	-2251.2	-73.6	-2177.6	-3905, -451	**0.018**
Mediterraneibacter	-436.2	136.7	-572.9	-1866, 720	0.39	11.3	1286.6	-1275.3	-3328, 777	0.23
Agathobacter	127.3	222.2	-94.9	-983, 793	0.84	-497.4	268.5	-765.9	-2194, 662	0.30
Agathobaculum	-409.7	-142.5	-267.3	-1631, 1096	0.70	-1368.7	487.3	-1856	-4009, 297	0.09
Lachnospiraceae NK4A136 group	647.3	-358.7	1006.0	334, 1678	**0.0055**	192.4	-434.6	627	-536, 1790	0.29
**POST Abundance**										
Streptococcus	-118.0	99.4	-217.4	-1153, 718	0.65	-730.5	-152.5	-578.0	-2126, 970	0.47
Lachnoclostridium	-145.8	762.1	-907.8	-1723, -92	**0.03**	-1173.8	28.8	-1202.6	-2646, 241	0.11
Mediterraneibacter	-191.2	919.7	-1110.9	-2307, 85	0.08	-539.6	146.7	-686.3	-2609, 1236	0.48
Agathobacter	420.5	-52.7	473.2	-452, 1399	0.32	-507.4	478.25	-985.7	-2442, 470	0.19
Agathobaculum	-285.9	-221.0	-64.9	-1190, 1060	0.91	-1355.7	81.7	-1437.4	-3254, 379	0.13
Lachnospiraceae NK4A136 group	-146.2	-718.52	572.3	-148, 1293	0.13	-250.5	128.6	-379.1	-1626, 868	0.55

[1] β values represent the change in outcome per 1-unit increase in CLR-transformed taxon abundance. β_{WHEAT} and β_{WHITE} represent diet-specific simple slopes derived from linear mixed-effects models. β_{int} is the β-coefficient interaction (WHEAT – WHITE). CI_{int} is the confidence interval for the β-coefficient interaction. P_{int} refers to the p-value for the interaction effect (CLR-abundance x treatment) of the linear mixed model.
